# Desulfation of Heparan Sulfate by Sulf1 and Sulf2 Is Required for Corticospinal Tract Formation

**DOI:** 10.1038/s41598-017-14185-3

**Published:** 2017-10-23

**Authors:** Takuya Okada, Kazuko Keino-Masu, Satoshi Nagamine, Fuyuki Kametani, Tatsuyuki Ohto, Masato Hasegawa, Toin H. van Kuppevelt, Satoshi Kunita, Satoru Takahashi, Masayuki Masu

**Affiliations:** 1 Department of Molecular Neurobiology, Faculty of Medicine, University of Tsukuba, 1-1-1 Tennodai, Ibaraki, 305-8575 Japan; 2 Department of Pediatrics, University of Tsukuba Hospital, 2-1-1 Amakubo, Ibaraki, 305-8576 Japan; 3 Laboratory Animal Resource Center, University of Tsukuba, 1-1-1 Tennodai, Tsukuba, Ibaraki, 305-8575 Japan; 4 Department of Neuropathology and Cell Biology, Tokyo Metropolitan Institute of Medical Science, Setagaya-ku, Tokyo, 156-8506 Japan; 5 Department of Biochemistry, Nijmegen Institute for Molecular Life Sciences, Radboud University Medical Center, Nijmegen, The Netherlands; 6 Pharmaceuticals and Medical Devices Agency, 3-3-2 Kasumigaseki, Chiyoda-Ku, Tokyo, 100-0013 Japan; 7 Present Address: Center for Experimental Medicine, Jichi Medical University, 3311-1 Yakushiji, Shimotsuke, Tochigi, 329-0498 Japan

## Abstract

Heparan sulfate (HS) has been implicated in a wide range of cell signaling. Here we report a novel mechanism in which extracellular removal of 6-*O*-sulfate groups from HS by the endosulfatases, Sulf1 and Sulf2, is essential for axon guidance during development. In *Sulf1/*2 double knockout (DKO) mice, the corticospinal tract (CST) was dorsally displaced on the midbrain surface. *In utero* electroporation of *Sulf1/*2 into radial glial cells along the third ventricle, where *Sulf1/*2 mRNAs are normally expressed, rescued the CST defects in the DKO mice. Proteomic analysis and functional testing identified Slit2 as the key molecule associated with the DKO phenotype. In the DKO brain, 6-*O*-sulfated HS was increased, leading to abnormal accumulation of Slit2 protein on the pial surface of the cerebral peduncle and hypothalamus, which caused dorsal repulsion of CST axons. Our findings indicate that postbiosynthetic desulfation of HS by Sulfs controls CST axon guidance through fine-tuning of Slit2 presentation.

## Introduction

Heparan sulfate (HS) is a polysaccharide attached to the core poteins of proteoglycans present in the extracellular matrix (ECM) and on the cell surface^1–4^. It interacts with growth factors, morphogens, and their receptors, thereby regulating their distribution and signal transduction. HS consists of repeating disaccharides, each of which is composed of hexuronic acid (glucuronic acid or iduronic acid) and *N*-acetylglucosamine. During biosynthesis, its sugar backbone undergoes extensive sulfation at some of the 2-*O*-positions of iduronic acids and the 3-*O*-, 6-*O*-, and *N*-positions of glucosamine residues. Because sulfation patterns of the sugar chain determine whether HS binds to ligands and how strong the binding is, biochemical studies of HS sulfation by sulfotransferases during biosynthesis have been the central issue in understanding the functional roles of HS.

This classical view was revised by the discovery of the endosulfatases, Sulfatase 1 (Sulf1) and Sulfatase 2 (Sulf2), which selectively remove 6-*O*-sulfate groups from mature HS in the extracellular space after HS biosynthesis is completed^5,6^. They preferentially act on trisulfated disaccharides possessing sulfate groups at the 2-*O*-, 6-*O*-, and *N*-positions in HS. Through 6-*O*-desulfation, Sulfs regulate multiple signaling pathways positively or negatively^5,6^. Their physiological roles have been uncovered by analyzing *Sulf* knockout (KO) mice: single KO mice appear largely normal, whereas double knockout (DKO) mice show multiple defects in growth, development, and regeneration^7–13^. However, the roles of *Sulfs* in neural circuit formation *in vivo* have yet to be elucidated.

In this study, we report that *Sulf1/2* DKO mice have axon guidance defects in the corticospinal tract (CST). We provide evidence that abnormal accumulation of Slit2 protein, caused by increases in 6-*O*-sulfated HS, leads to axonal defects. Our findings demonstrate that Sulf-mediated HS desulfation in the ECM controls CST axon guidance through regulating the appropriate Slit2 presentation.

## Results

### *Sulf1* and *Sulf2* Play a Role in Generating Sulfation Patterns of HS *In Vivo*

To assess whether the sulfation patterns of HS are changed in *Sulf* KO brains, we first performed HS disaccharide analysis. Because *Sulf1/2* DKO mice died within a day of birth for unknown reasons, we used neonatal mice. HS was digested into disaccharides by heparin lyases, and the compositions of 8 different disaccharides possessing sulfate residues at different combinations at the 2-*O*-, 6-*O*-, and *N*-positions were determined by HPLC analysis. The *Sulf1* KO and *Sulf2* KO brains showed increases in 2-*O*-, 6-*O*-, and *N*-sulfated disaccharides and decreases in 2-*O*- and *N*-sulfated disaccharides (Supplementary Fig. S1a–b). In the *Sulf1/2* DKO brains, the changes were more than a simple additive effect of the two single mutants (Supplementary Fig. S1a–b), indicating redundant roles of Sulf1 and Sulf2. The sulfation profiles of chondroitin sulfate were not changed in the mutant brains (data not shown). These results demonstrate that *Sulf1* and *Sulf2* play a cooperative role in generating the sulfation patterns of HS in neonatal brains.

### *Sulf1/2* DKO Mice Have Defects in CST Axon Guidance

**Figure 1:**
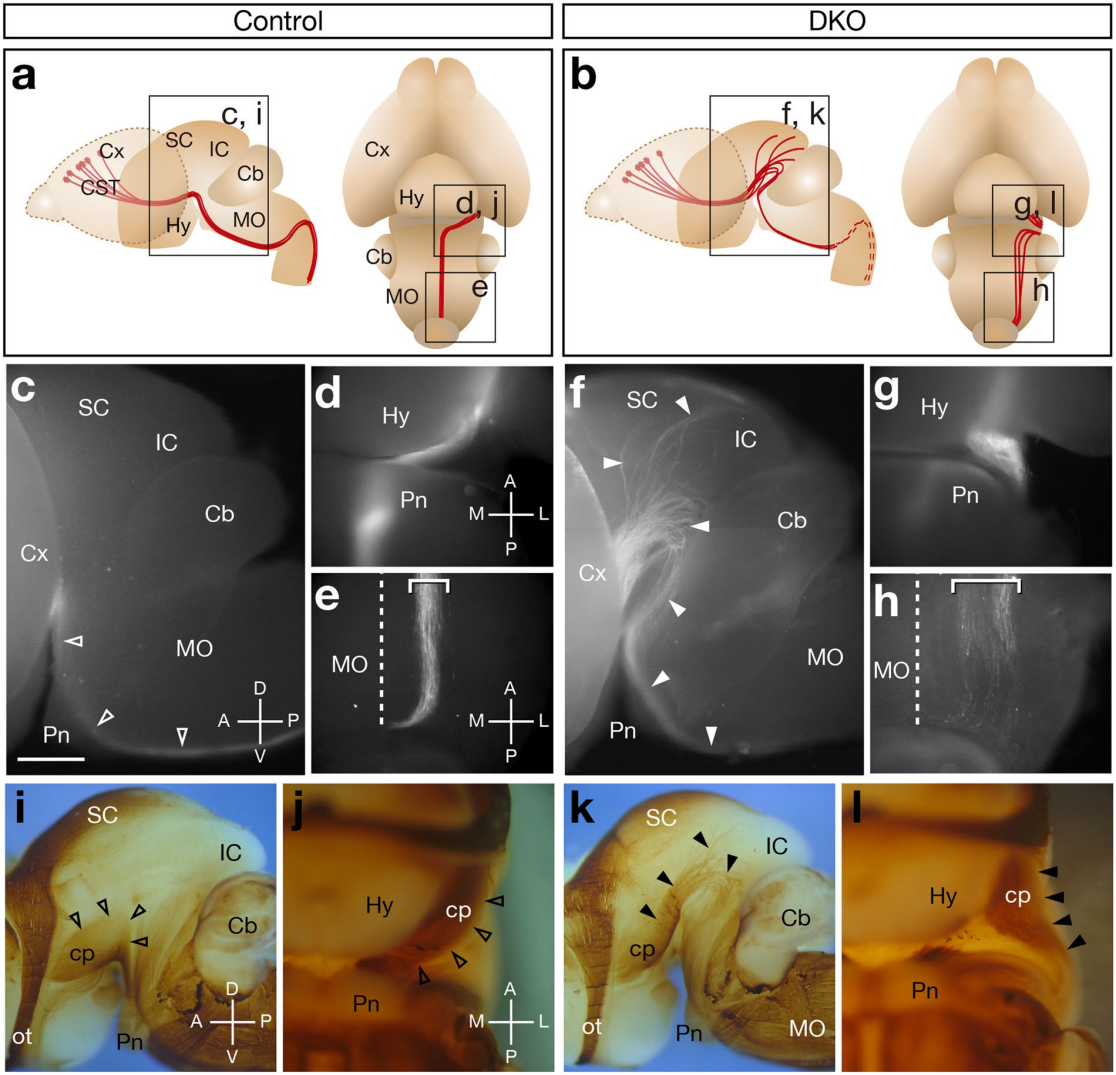
CST Axon Guidance Defects in *Sulf1/2* DKO Mice. (**a**,**b**) Trajectory of the CST. Boxes show the areas of the pictures in (**c**–**l**). (**c**–**h**) Fluorescence images of P0 brains injected with DiI in the motor cortices. Lateral (**c**,**f**) and ventral (**d**,**e**,**g**,**h**) views of the control (*Sulf1* ^−/−^;*Sulf2* ^+/−^; **c**–**e**) and *Sulf1/2* DKO brains (**f**–**h**) are shown. *Sulf1/2* DKO mice showed abnormal defasciculated axons (closed arrowheads in **f**, box in **b**). The dashed lines indicate the midline. The average intensity of fluorescence signals in the midbrain area was significantly higher in the DKO mice than that in the control mice (18.388 arbitrary unit in control and 29.096 in DKO; n = 4, *P* = 0.018637 by Welch’s t-test). (**i**–**l**) Whole-mount neurofilament staining of the E18.5 brain. Lateral views (**i**,**k**) and ventral views (**j**,**l**) of control (*Sulf1* ^−/−^; **i**–**j**) and *Sulf1/2* DKO brains (**k**,**l**) are shown. The cerebral cortices were removed. Abnormal fibers (closed arrowheads) were observed in the *Sulf1/2* DKO brain (**k**,**l**). Open arrowheads in (**i**,**j**) indicate the normal cerebral peduncle. Statistical analyses of the neurofilament-positive fibers in the midbrain (**i**,**k**) are shown in Supplementary Table 1. Cb, cerebellum; cp, cerebral peduncle; Cx, cerebral cortex; Hy, hypothalamus; IC, inferior colliculus; MO, medulla oblongata; ot, optic tract; Pn, pons; SC, superior colliculus. Anterior-posterior (A-P), dorsal-ventral (D-V), and medial-lateral (M-L) body axes are shown. Scale bars indicate 750 μm (**c**,**f**), 500 μm (**d**,**e**,**g**,**h**), 1.0 mm (**i**,**k**), and 600 μm (**j**,**l**), See also Supplementary Fig. S1.

Because no gross brain malformations were observed, we performed intensive histological searches for possible abnormalities in the nerve tracts of *Sulf1/2* DKO mice. Consequently, we found that the cerebral peduncle was aberrant and the pyramidal tract was reduced in size in neurofilament-M-stained brain sections (Supplementary Fig. S1n–o), indicating that the *Sulf1/2* DKO mice had defects in the CST. The CST originates in layer 5 of the sensorimotor cortex, descends through the internal capsule, cerebral peduncle, and ventral medulla, and projects to the spinal cord postnatally^14,15^. To selectively examine the trajectory of the CST axons, we performed DiI tracing. When DiI was injected into the motor cortex of live neonatal mice, CST axons were labeled up to the pyramidal decussation within 10 h (Fig. 1a,c–e).

In the DKO mice, most of the labeled fibers extended dorsally towards the superior colliculus and then returned to the brainstem, whereas some fibers invaded the superior and inferior colliculi (Fig. 1b,f). The fibers that returned to the medulla were defasciculated and positioned more laterally (Fig. 1b,h), which was in contrast to the tightly fasciculated bundle in the control mice **(**Fig. 1a,e). As a result, the width of the CST axon bundles in the medulla was significantly greater in the DKO mice than that in the control mice (202.6 μm in control and 374.4 μm in DKO, n = 4; *P* = 0.002635 by Welch’s t-test). Section analysis of the DiI-injected brains showed that the CST fibers of the DKO mice appeared to be almost normal until they reached the midbrain, where the misdirected fibers extended dorsolaterally along the brain surface (Supplementary Fig. S1p–t). In the medulla of the DKO mice, the pyramidal tract became thinner and broader (Supplementary Fig. S1u). These abnormalities were consistent with the defects detected by the previous neurofilament-M staining (Supplementary Fig. S1m–o). Additional defects were observed inside the DiI-injected brains: a small portion of the cortical fibers projected aberrantly towards the tectum through the thalamus (n = 4/4, Supplementary Fig. S1r) and some mice showed midline-crossing defects in the corpus callosum (n = 1/4, data not shown).

Because the striking abnormalities of the CST axons were present on the brain surface and could be detected by neurofilament antibody, we performed whole-mount neurofilament-M staining at embryonic day 18.5 (E18.5), when the CST axons reach the medulla. In the control brain, the CST axons were seen as a large ventral bundle (the cerebral peduncle): they emerged onto the brain surface from the posterolateral side of the hypothalamus and immediately turned medially towards the pons (Fig. 1a,i–j). In contrast, in the *Sulf1/2* DKO mice, the CST axons extended dorsally and were defasciculated on the lateral surface of the midbrain (Fig. 1b,k). When viewed from the ventral aspect, the CST fibers turned laterally after exiting the hypothalamus (Fig. 1b,l). Thus, this method can assess the CST defects in DKO mice clearly.

Because whole-mount neurofilament staining is more useful to examine the overall changes in axonal trajectories on the brain surface without experimental bias and because many samples can be evaluated without sectioning, we adopted this method in the subsequent experiments to determine the presence of CST abnormalities. When a number of *Sulf1/2* DKO mice were examined, the CST defects were somewhat variable between individuals. Most of the DKO mice showed an aberrant CST trajectory in the midbrain, as shown in Fig. 1k,l (62 of 70 tracts examined, Supplementary Fig. S1y and S1y’). In one severe case, all the CST axons turned dorsally and projected to the superior colliculus (1/70, Supplementary Fig. S1z and S1z’). In less severe cases, the CST axons turned medially towards the pons after exiting the hypothalamus but were located lateral to the medial lemniscus (7/70, Supplementary Fig. S1x and S1x’), whereas they overrode the medial lemniscus in the control brains (Supplementary Fig. S1w’). In summary, all the *Sulf1/2* DKO mice showed CST axon guidance defects, whereas no CST abnormalities were observed in the *Sulf1* ^−/−^, *Sulf2* ^−/−^, *Sulf1* ^−/−^;*Sulf2* ^+/−^, or *Sulf1* ^+/−^;*Sulf2* ^−/−^ mice (data not shown). We thus focused on this robust defect and explored the underlying mechanism.

### Electroporation of *Sulf* Genes Into the Hypothalamus and Midbrain Rescues the CST Defects in *Sulf1/2* DKO Mice

**Figure 2:**
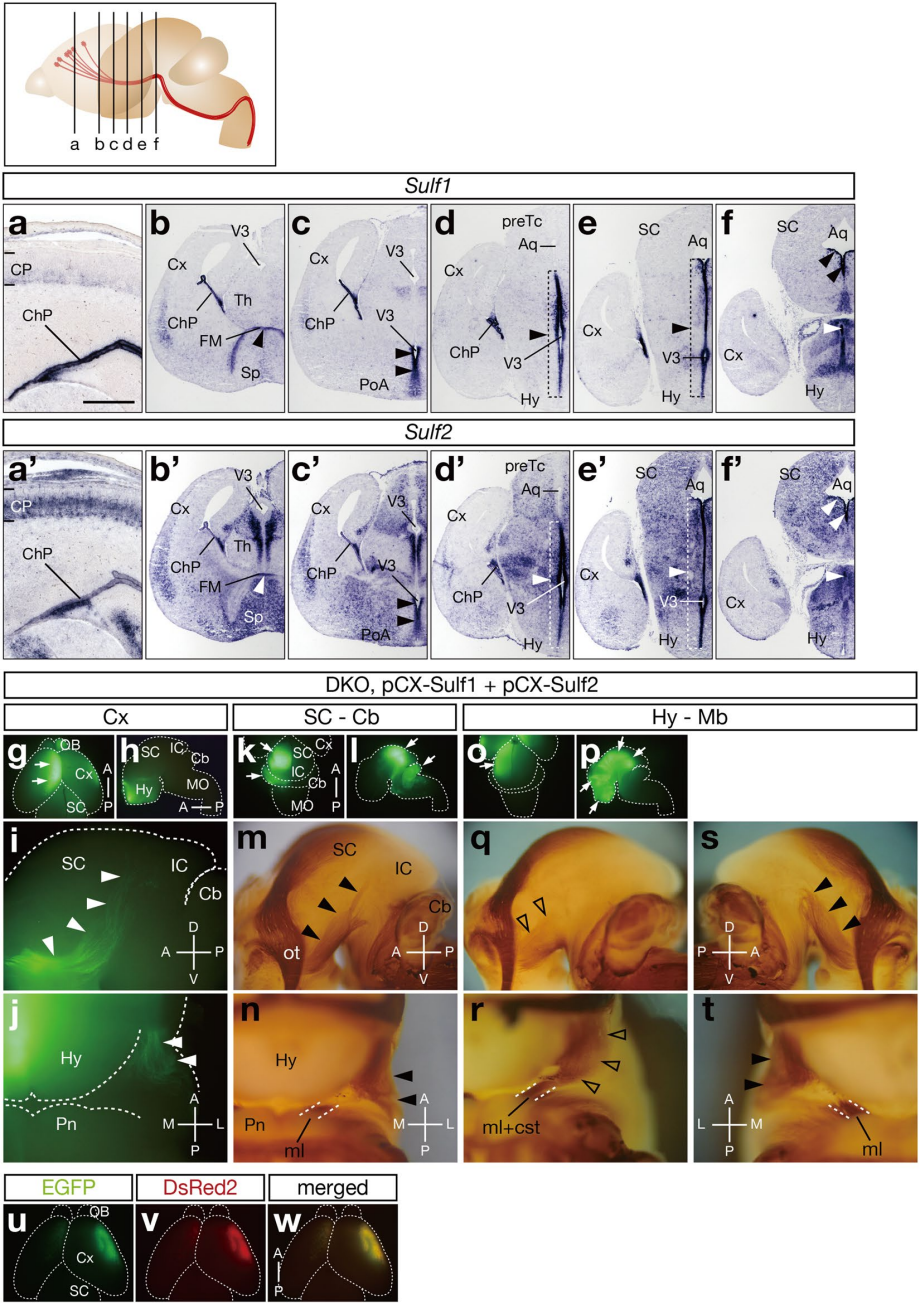
Expression of *Sulf1* and *Sulf2* mRNAs and *In Vivo* Rescue of the CST Defects in *Sulf1/2* DKO Mice. (**a**–**f**,**a’**–**f’**) *In situ* hybridization of *Sulf1* (**a**–**f**) and *Sulf2* (**a’**–**f’**) in the coronal sections of E15.5 brains. Arrowheads show high and overlapping expression of *Sulf1* and *Sulf2* in the ventricular zone of the third ventricle and aqueduct. Positions of the sections in (**a**–**f**) and (**a’**–**f’**) are shown in the upper margin. (**g**–**t**) Electroporation-mediated rescue of the CST defects in *Sulf1/2* DKO mice. The indicated plasmids and pCX-*EGFP* were electroporated into E12.5 *Sulf1/2* DKO brains. At E18.5, EGFP expression and the CST trajectory were examined. Representative results showing electroporation into the medial cerebral cortex (**g**–**j**), from the superior colliculus to the cerebellum (**k**–**n**), and from the hypothalamus to the midbrain (Mb, **o**–**t**) are shown. Figures (**q**,**r**) and (**s**,**t**) show the electroporated and nonelectroporated sides of the same embryo, respectively. Dorsal (**g**,**k**,**o**), lateral (**h**,**i**,**l**,**m**,**p**,**q**,**s**), and ventral (**j**,**n**,**r**,**t**) views are shown. EGFP fluorescence in the hypothalamus in (**h**) shows transmission through the contralateral side. Arrows indicate the sites of electroporation. Closed arrowheads (**i**,**j**,**m**,**n**,**s**,**t**) indicate abnormal CST fibers in the *Sulf1/2* DKO brain, whereas open arrowheads (**q**,**r**) indicate the CST restored by electroporation. Statistical analyses of the neurofilament-positive fibers in the midbrain (**q**,**s**) are shown in Supplementary Table 1. (**u**–**w**) Colocalization of coelectroporated genes. When *EGFP* and *DsRed2* were electroporated together, they were expressed in the same brain regions. Aq, aqueduct; ChP, choroid plexus; CP, cortical plate; cst, corticospinal tract; FM, foramen of Monro; ml, medial lemniscus; OB, olfactory bulb; PoA, preoptic area; preTc, pretectum; Sp, septum; Th; thalamus; V3, third ventricle. Anterior-posterior (A-P), dorsal-ventral (D-V), and medial-lateral (M-L) body axes are shown. Scale bars indicate 300 μm (**a**,**a’**), 750 μm (**b**–**f**,**b’**–**f’**), 3.1 mm (**g**,**h**,**k**,**l**,**o**,**p**), 550 μm (**i**,**j**,**n**,**r**,**t**), 1.0 mm (**m**,**q**,**s**), and 2.1 mm (**u**–**w**). See also Supplementary Fig. S2.

To examine the *Sulf* expression potentially relevant to CST development, we performed *in situ* hybridization. Because CST axons pass through the cerebral peduncle at E15 and reach the medulla at E17–18 in mice^14,15^, we examined *Sulf* expression at E15.5. *Sulf1* mRNA showed relatively restricted expression in the choroid plexus, the cortical hem, and the ventricular zone of the third ventricle (Fig. 2a–f), whereas *Sulf2* mRNA showed broader expression in the brain (Fig. 2a’–f’). In the cortical plate, *Sulf1* and *Sulf2* mRNAs were observed in the presumptive layer 6 and in layers 5–6, respectively (Fig. 2a and a’). Outside the cerebral cortex, *Sulf1* and *Sulf2* showed strong and overlapping expression in the ventricular zone of the third ventricle and aqueduct (Fig. 2c–f and 2c’–f’).

To determine which brain regions of *Sulf* expression are required for CST formation, we performed *in vivo* rescue of the *Sulf1/2* DKO phenotype by local introduction of *Sulf* genes. For this purpose, we electroporated *Sulf1* and *Sulf2* with *EGFP* into various brain areas at E12.5 and examined the EGFP-positive area and CST trajectory at E18.5. We performed electroporation at E12.5 because it was before the CST axons began to extend and because the ventricles of the embryonic brains were widely open, enabling easy introducion of exogenous genes into various regions. To induce strong and ubiquitous expression, we used a pCX vector carrying the CAG promoter^16^. By holding embryos with electrodes at different angles, exogenous genes could be electroporated into restricted brain regions of interest (Fig. 2g–t). Because the coelectroporated genes were coexpressed in the same brain region (Fig. 2u–w and Supplementary Fig. S2a–d), the EGFP-positive areas represented the brain regions where *Sulf* genes had been introduced.

Electroporation of pCX-*Sulf1/2* into the cerebral cortex (5 mice; Fig. 2g–j) and into the area from the superior colliculus to the cerebellum (3 mice; Fig. 2k–n) failed to rescue the DKO phenotype. In contrast, when *Sulf1*/*2* were electroporated into the area including the hypothalamus and midbrain, the CST defects were completely restored to normal only on the electroporated side (6 mice; Fig. 2o–t). Moreover, a mutant *Sulf1* lacking enzyme activity could not rescue the phenotype (4 mice; Supplementary Fig. S2e–h), whereas wild-type *Sulf1* or *Sulf2* could rescue the defects (4 mice for *Sulf1* and 3 mice for *Sulf2*; Supplementary Fig. S2i–p). These data indicate that Sulf activities are required not in the cortical axons, but in the middle of the CST trajectory.

### *Sulf* Expression in the Radial Glial Cells Along the Third Ventricle Is Required for Navigating CST Axons

**Figure 3:**
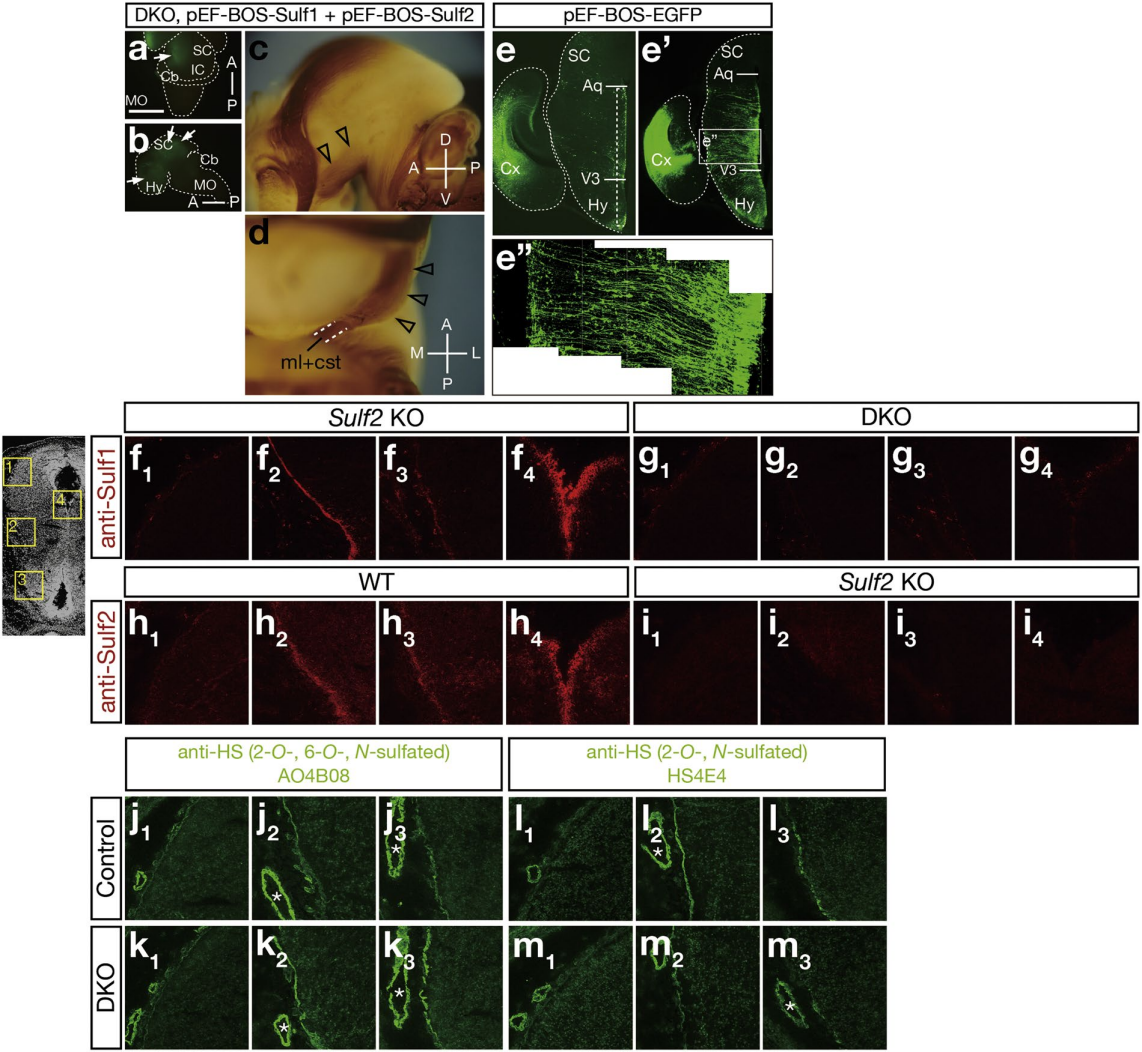
Electroporation of *Sulf* Genes Into the Radial Glial Cells in the Hypothalamus Restores the CST Defects in *Sulf1/2* DKO Mice. (**a**–**d**) Electroporation-mediated rescue of the CST defects in *Sulf1/2* DKO mice. The indicated plasmids and pEF-BOS-*EGFP* were electroporated into E12.5 *Sulf1/2* DKO brains. At E18.5, EGFP expression and the CST trajectory were examined. Dorsal (**a**), lateral (**b**,**c**), and ventral (**d**) views are shown. Open arrowheads (**c**,**d**) indicate the CST restored by electroporation. Arrows indicate the sites of electroporation. Anterior-posterior (A-P), dorsal-ventral (D-V), and medial-lateral (M-L) body axes are shown. Statistical analyses of the neurofilament-positive fibers in the midbrain (**c**) are shown in Supplementary Table 1. (**e**–**e”**) EGFP expression in pEF-BOS-*EGFP*-electroporated brains. (**e’**) shows the immunohistochemistry with anti-EGFP antibody. (**e”**) shows the magnified picture in the boxed region in (**e’**). (**f**–**m**) Immunohistochemistry of the E15.5 brain with anti-Sulf or anti-HS antibodies. The signals with anti-Sulf1 in the *Sulf2* KO brain (**f**) were abolished in the *Sulf1/2* DKO brain (**g**), whereas the signals with anti-Sulf2 in the wild-type brain (**h**) were abolished in the *Sulf2* KO (**i**) brain. *Sulf2* KO and *Sulf1/2* DKO brains were used for anti-Sulf1 staining because anti-Sulf1 antibody weakly crossreacts with Sulf2 protein. The signals with anti-HS AO4B08 (**j**,**k**) and anti-HS HS4E4 (**l**,**m**) in the control (*Sulf1* ^−/−^) brain (**j**,**l**) were increased and decreased in the *Sulf1/2* DKO brain (**k**,**m**), respectively. Pictures (**f**_**1**_–**m**_**1**_), (**f**_**2**_–**m**_**2**_), (**f**_**3**_–**m**_**3**_), and (**f**_**4**_–**i**_**4**_) show the boxed regions with the corresponding numbers in the brain section in the left margin. Asterisks show blood vessels. Scale bars indicate 2.0 mm (**a**,**b**), 650 μm (**c**,**e**,**e’**), 350 μm (**d**), 210 μm (**e”**), and 150 μm (**f**–**m**). See also Supplementary Fig. S2.

Because the CST defects were observed only when all 4 *Sulf* alleles were lost, it is plausible that the brain areas expressing both *Sulf1* and *Sulf2* are associated with the defects. Given that electroporation of *Sulf* genes into the hypothalamus and the midbrain rescued the DKO phenotype, it is likely that strong overlapping expression of *Sulf1*/*2* in the ventricular zone of the third ventricle plays a critical role. We thus wondered whether selective electroporation of *Sulf* genes into these cells could rescue the DKO phenotype. Conveniently, we found that the pEF-BOS vector^17^ can induce relatively specific expression in these cells, although the underlying mechanism is unknown. When electroporated into the hypothalamus and midbrain, pEF-BOS-*EGFP* induced weak but restricted expression in the cells in the ventricular zone (Fig. 3a,b,e), whereas pCX-*EGFP* induced widespread expression (Fig. 2o,p and Supplementary Fig. S2a,c). We therefore electroporated pEF-BOS-*Sulf1* and pEF-BOS-*Sulf2* into the *Sulf1/2* DKO brain and found that the CST defects were completely rescued (3 mice; Fig. 3c,d). These data indicate that *Sulf* expression in the ventricular zone of the third ventricle was sufficient to steer CST axons properly.

To examine Sulf protein localization in the brain, we performed immunostaining. Both Sulf1 and Sulf2 proteins were detected in the cells in the ventricular zone along the third ventricle (Fig. 3f_4_,h_4_), consistent with the localization of *Sulf1* and *Sulf2* mRNA. Both signals were abolished by disruption of *Sulf* genes, indicating the specificity of the immunostaining (Fig. 3g_4_,i_4_). Interestingly, both proteins were additionally detected on the brain surface: strongly near the cerebral peduncle and weakly in the hypothalamus (Fig. 3f_2–3_,h_2–3_). Sulf proteins induced by electroporation displayed similar distribution patterns (Supplementary Fig. S2b_3_,d_3_). Because EGFP-positive cells in the pEF-BOS-EGFP-electroporated brain were radial glial cells extending long processes from the ventricular zone to the pial surface (Fig. 3e’–3e”), we surmised that Sulf proteins produced by radial glial cells are delivered to the pial surface.

To test whether disruption of *Sulf* genes alters HS sulfation patterns locally where Sulf proteins are present, we performed immunostaining of HS using several anti-HS phage display antibodies at E15.5, even though our disaccharide analysis had already revealed that trisulfated HS disaccharides were increased in the whole brains of the neonatal DKO mice (Supplementary Fig. S1a,b). The signals with AO4B08 and RB4CD12 antibodies, which recognize HS disaccharides containing the 2-*O*-, 6-*O*-, and *N*-sulfate groups^18,19^, were stronger on the brain surface of the DKO mice than on that of the control mice, whereas the signals in the blood vessels were comparable between the DKO and the control mice (Fig. 3j,k and Supplementary Fig. S2q–r). The increases in AO4B08 and RB4CD12 signals were particularly prominent in the cerebral peduncle and hypothalamus. Conversely, the signals with HS4E4, which recognizes HS disaccharides containing the 2-*O*- and *N*-sulfate groups^18^, were weaker in the DKO mice (Fig. 3l,m). These findings indicate that the proportion of trisulfated HS disaccharides was increased on the pial surface as a result of *Sulf1/2* disruption. Because the changes were most robust around the CST trajectory, it is likely that this local change in HS contributes to the emergence of abnormalities in CST axons.

### *Slit* Overexpression Mimics the Axon Guidance Defects in *Sulf1/2* DKO Mice

What are the mechanisms underlying the CST defects in *Sulf1/2* DKO mice? We hypothesized that increases in trisulfated HS altered the amount or localization of some axon guidance protein(s). To determine the responsible molecule(s), we first investigated all the known axon guidance proteins expressed in the hypothalamus and midbrain at E15.5 by an unbiased approach using proteomic analysis. Because Sulfs modified HS in the basement membrane, the meninges (enriched in the basement membrane) were analyzed using liquid chromatography-ion trap mass spectrometry (LC-MS/MS). The table in Supplementary Fig. S3a shows the list of axon guidance proteins detected by this method. Slit2, Sema3E, Sema4G, Sema5B, and Sema6B were detected only in the DKO brain, suggesting that they were more abundant in the DKO mice. In contrast, Sema4D and EphrinB2 were detected only in the control brain, suggesting that they were less abundant in the DKO mice. Slit1 showed the same spectral counts in the control and the DKO mice.

**Figure 4:**
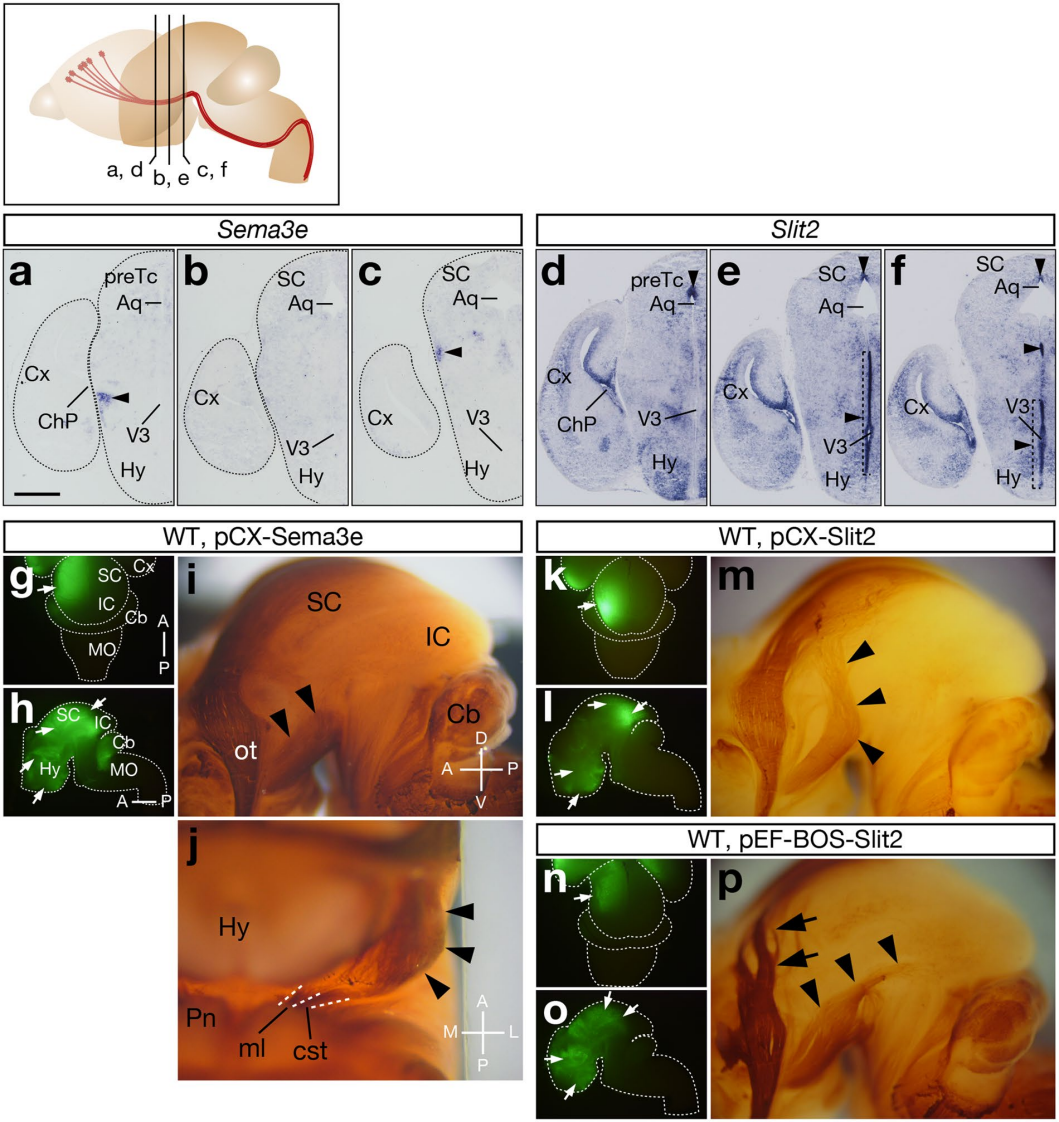
Slit2 and Sema3e Overexpression Causes the CST Defects. (**a**–**f**) *In situ* hybridization of *Sema3e* and *Slit2* in coronal sections of the E15.5 brain. *Sema3e* was expressed near the cerebral peduncle (**a**–**c**, arrowheads), whereas *Slit2* was highly expressed in the ventricular zone of the posterior hypothalamus and the dorsal midline of the aqueduct (**d**–**f**, arrowheads). Positions of the sections in (**a**–**f**) are shown in the upper margin. (**g**–**p**) Effects of *Sema3e* and *Slit2* overexpression on CST axons. The indicated plasmids and pCX-*EGFP* were electroporated into wild-type brains at E12.5 (**g**–**m**) or E13.0 (**n**–**p**). At E18.5, EGFP expression and the CST trajectory were examined. *Sema3e* overexpression induced lateral shift of the CST (**i**,**j**, arrowheads), which was similar to the mild phenotype of *Sulf1/2* DKO mice. Strong *Slit2* overexpression by pCX-*Slit2* electroporation strongly repelled CST axons (**m**, arrowheads), which was similar to the severe phenotype of *Sulf1/2* DKO mice. Next, weaker *Slit2* overexpression was induced by electroporation of pEF-BOS-*Slit2* at E13.0, when electroporation efficiency decreased owing to the narrowing of the ventricles (compare the EGFP signals induced by pCX-*EGFP* electroporated at E12.5 [**4g**–**h** and **4k**–**l**] and at E13.0 [**4n**–**o**]). Mild *Slit2* expression thus obtained induced weaker CST defects (**p**, arrowheads), which was similar to the moderate phenotype of *Sulf1/2* DKO mice. Defects in retinotectal projection were also observed (**p**, arrows). Arrows in (**g**–**h**), (**k**–**l**), and (**n**–**o**) indicate the sites of electroporation. Anterior-posterior (A-P), dorsal-ventral (D-V), and medial-lateral (M-L) body axes are shown. Statistical analyses of the neurofilament-positive fibers in the midbrain (**i**,**p**) are shown in Supplementary Table 1. Scale bars indicate 550 μm (**a**–**f**), 1.8 mm (**g**,**h**,**k**,**l**,**n**,**o**), and 600 μm (**i**,**j**,**m**,**p**). See also Supplementary Fig. S3.

To pin down the candidate molecule(s), we examined their expression patterns and tested whether their overexpression induced any changes in the CST trajectory. Electroporation of pCX-*Sema3e* into wild-type brains induced lateral shift of the CST in the cerebral peduncle (3 mice, Fig. 4i,j), resembling the weak DKO phenotype. *Sema3e* mRNA was expressed near the cerebral peduncle (Fig. 4a–c), suggesting possible involvement in CST formation. However, *Sema3e* overexpression did not elicit such CST defects as observed in the *Sulf1/2* DKO brain, indicating that excess Sema3E alone cannot account for the DKO phenotype. Electroporation of pCX-*Slit2* at E12.5 caused very strong repulsion of the CST axons (Fig. 4m), similar to the severe DKO phenotype and consistent with previous observations^20,21^. Induction of weaker *Slit2* expression by electroporation of pEF-BOS-*Slit2* (pEF-BOS induces weaker expression than pCX) at E13.0 (lower electroporation efficiency than at E12.5) led to CST defects that were indistinguishable from those of the moderate DKO phenotype (Fig. 4p). *Slit2* mRNA showed high expression in the ventricular zone of the third ventricle, especially in the posterior hypothalamus (Fig. 4d–f and Supplementary Fig. S3p–s), suggesting colocalization and possible collaboration with *Sulf1*/*2*. By contrast, *Slit1* expression was not seen in the ventricular zone of the ventral third ventricle (Supplementary Fig. S3l–o), and therefore, *Slit1* was thought to be irrelevant to the CST defects although its overexpression can induce repulsion of CST axons (Supplementary Fig. S3t–u). Electroporation of other candidate genes did not induce any CST abnormalities in the wild-type brains, nor did it rescue the DKO phenotype (Supplementary Fig. S3c,e,g,i,k).

**Figure 5:**
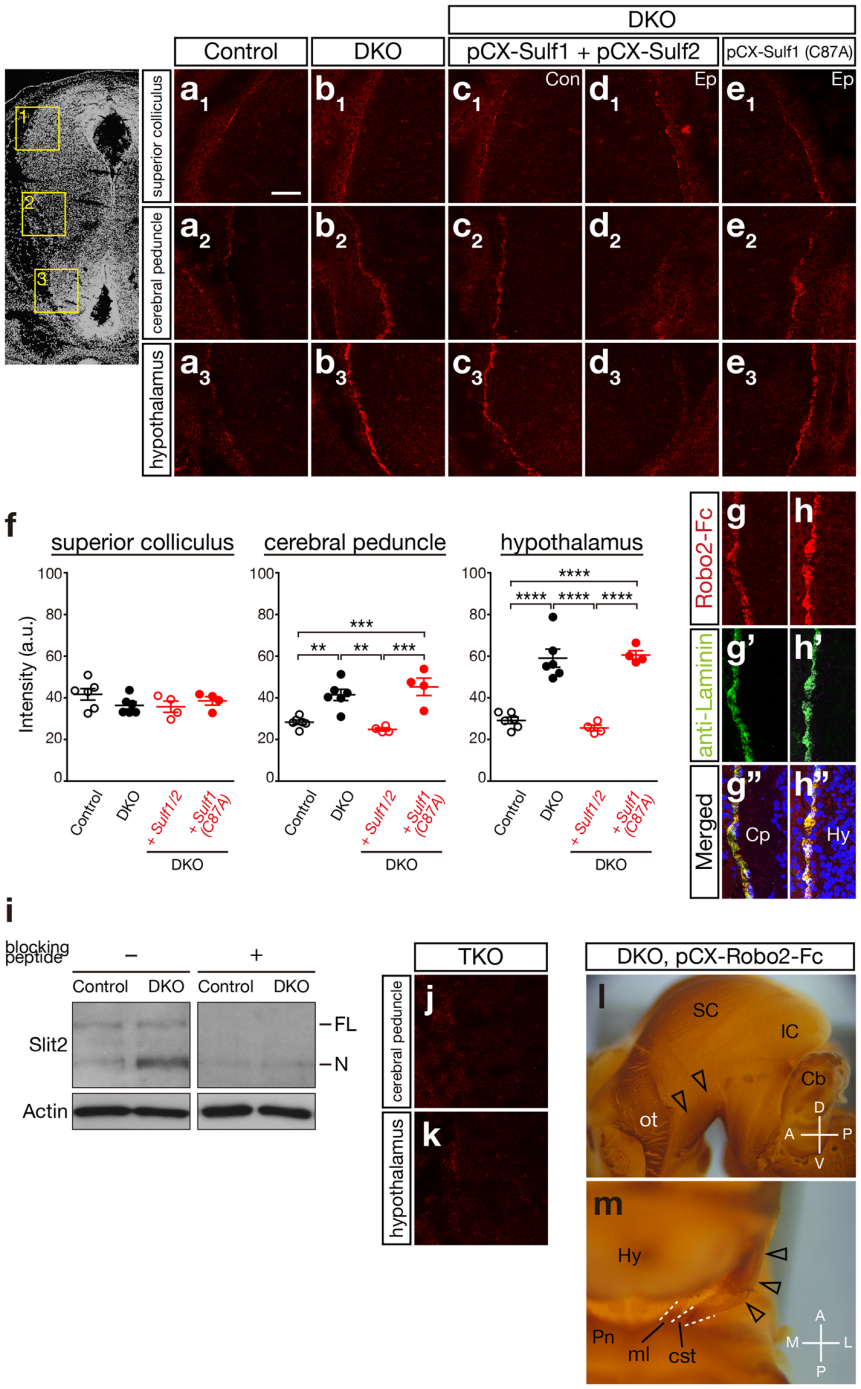
Slit2 Protein Accumulated Abnormally on the Pial Surface of the Posterior Hypothalamus of *Sulf1/2* DKO Mice. (**a**–**e**) Robo2-Fc binding on the brain sections of control (*Sulf1* ^−/−^) and *Sulf1/2* DKO mice and of *Sulf1/2* DKO mice electroporated with the indicated plasmids. (**a**_**1**_–**e**_**1**_), (**a**_**2**_–**e**_**2**_), and (**a**_**3**_–**e**_**3**_) indicate the areas including the superior colliculus, cerebral peduncle, and hypothalamus, respectively (shown in the boxed areas in the left panel). Figures (**a**–**c**) and Figures (**d**,**e**) show opposite sides of the brain sections. Con, control side; Ep, electroporated side. (**f**) Quantitative analysis of Robo2-Fc binding. Average of the fluorescence intensity (arbitrary unit; a.u.) on the pial surface obtained by confocal microscopy in control mice (n = 3, 2 *Sulf1* ^−/−^;*Sulf2* ^+/−^ and 1 *Sulf1* ^−/−^), *Sulf1/2* DKO mice (n = 3), and *Sulf1/2* DKO mice electroporated with pCX-*Sulf1/2* (n = 4) or pCX-*Sulf1*(*C87A*) (n = 4) are shown. Statistical significance was calculated using ANOVA with a Tukey-Kramer post hoc test (**P < 0.01; ***P < 0.001; ****P < 0.0001). (**g**,**h**) Colocalization of Robo2-Fc binding and anti-laminin immunostaining in the cerebral peduncle (**g**) and hypothalamus (**h**) of *Sulf1/2* DKO mice. (**i**) Western blot analysis of Slit2 protein in the meninges isolated from the hypothalamus and midbrain of E15.5 mice. Slit2 was more abundant in *Sulf1/2* DKO mice than in the control (*Sulf2* ^−/−^) mice. FL and N indicate the full-length (~180 kDa) and N-terminal fragments (~130 kDa), respectively. Addition of blocking peptide abolished the bands (right panel), indicating the specificity of the antibody. Full-length blots that were used for this picture are shown in Supplementary Fig. S7. (**j**,**k**) Robo2-Fc binding was completely abolished in the *Sulf1/Sulf2/Slit2* triple knockout (TKO) brain. (**l**,**m**) Neutralization of Slit rescues the CST defects in *Sulf1/2* DKO mice. The plasmids pCX-*Robo2-Fc* and pCX-*EGFP* were electroporated into E12.5 *Sulf1/2* DKO brains, and the CST trajectory was examined at E18.5. The CST was nearly normalized by electroporation (open arrowheads) but lay slightly lateral to the medial lemniscus (ml). Anterior-posterior (A-P), dorsal-ventral (D-V), and medial-lateral (M-L) body axes are shown. Scale bars indicate 100 μm (**a**–**e**,**j**,**k**), 50 μm (**g**,**h**), and 500 μm (**l**,**m**). See also Supplementary Fig. S4–7.

Therefore, Slit2 turned out to be the best candidate that accounted for the CST defects observed in the *Sulf1/2* DKO mice. Given that Slit2 binds to HS with high affinity^22^ in a 6-*O*-sulfate-dependent manner^23^, increased trisulfated HS is expected to bind more Slit2 protein in *Sulf1/2* DKO mice. Consistent with this prediction, the N-terminal fragment (an active form) of Slit2 protein was more abundant in the meninges of the DKO mice than in those of the control mice (Fig. 5i and Supplementary Fig. S4s). We thus hypothesized that increases in Slit2 protein in the ventral brain region caused dorsal repulsion of the CST axons in the DKO mice.

### Abnormally High Amounts of Slit2 Protein Accumulate on the Pial Surface of *Sulf1/2* DKO Mice

To test this possibility, we wished to detect Slit2 protein in embryonic brains. However, because no antibodies for immunostaining Slit2 were available, we used the extracellular portion of Robo2, a Slit receptor, which was tagged with the Fc region of human IgG (Robo2-Fc; ref.^24^). First, we confirmed that Robo2-Fc bound to Slit2 in the transfected cells (Supplementary Fig. S4a–d,f). Next, we tested Robo2-Fc binding on brain sections that were electroporated with pCX-FLAG-*Slit2*. When the brain sections were incubated with Robo2-Fc, the binding was clearly detected only on the electroporated side and was colocalized with the FLAG epitope (Supplementary Fig. S4m–r), indicating that Robo2-Fc can detect Slit2 protein in brain sections. Robo2-Fc binding was strongly detected on the brain surface in addition to the cell bodies of the electroporated cells in the ventricular zone and brain parenchyma.

We then tested whether the disruption of *Sulf* genes altered Slit localization. Robo2-Fc binding was hardly detectable in the control brains except for weak signals on the pial surface of the superior colliculus (Fig. 5a). In contrast, in *Sulf1/2* DKO mice, strong Robo2-Fc binding was detected on the pial surface, especially in the areas of the cerebral peduncle and hypothalamus (Fig. 5b). Quantitative comparison revealed that the signal intensity in the *Sulf1/2* DKO mice was significantly higher in the cerebral peduncle and hypothalamus than that in the controls but was comparable in the superior colliculus (Fig. 5f). Robo2-Fc binding was colocalized with laminin (Fig. 5g–h), suggesting that Slit protein was associated with the basement membrane. Furthermore, Robo2-Fc binding in the DKO brain was completely abolished by deletion of the *Slit2* gene (namely in the triple KO brain), except for the weak signals in the superior colliculus (Fig. 5j–k and Supplementary Fig. S5a–e), suggesting that the Robo2-Fc binding signals in the cerebral peduncle and hypothalamus are indicative of Slit2 protein localization. These data clearly showed that Slit2 protein accumulated excessively in the ventral brain region of *Sulf1/2* DKO mice.

**Figure 6:**
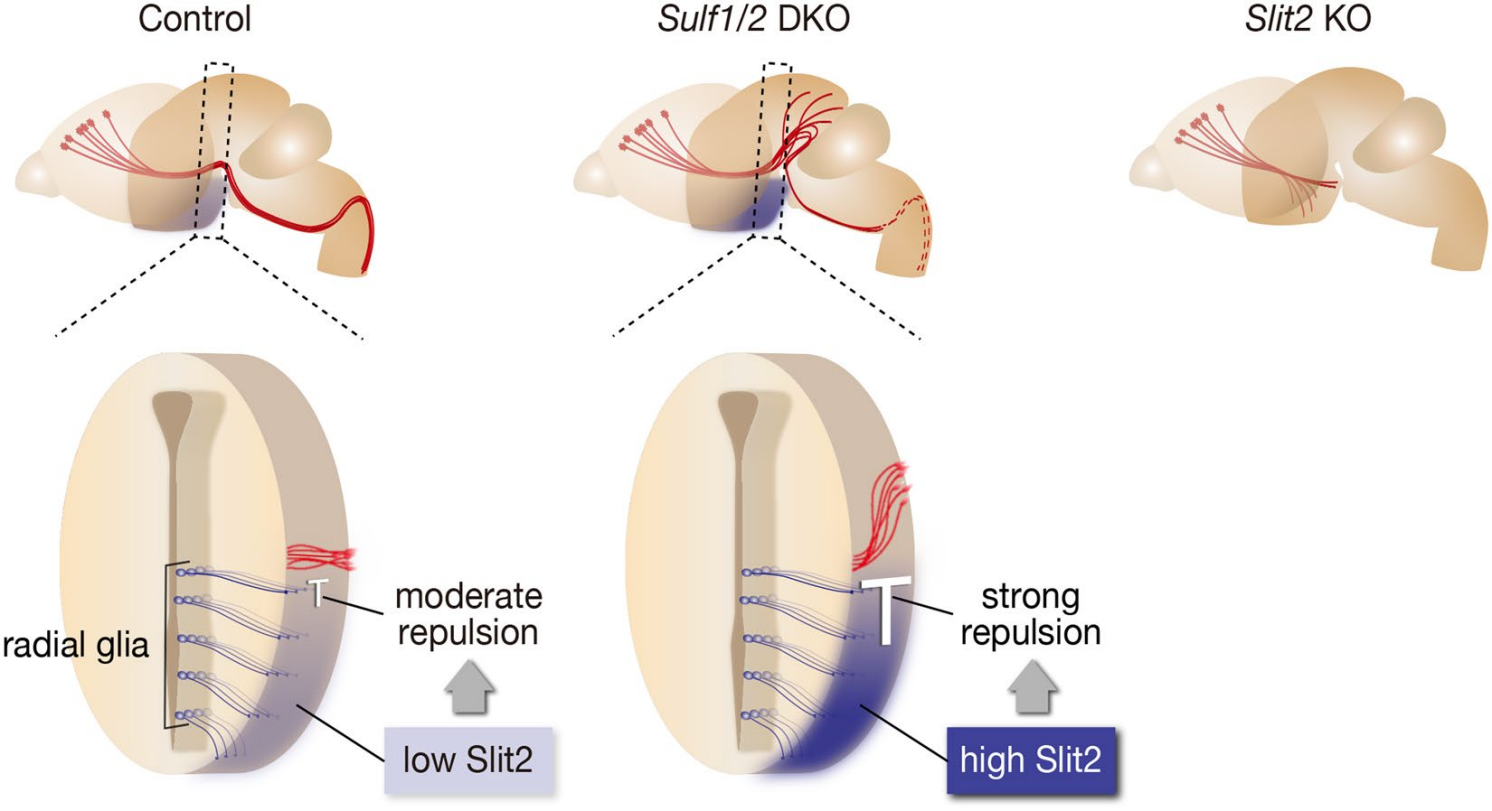
Summary of the CST Phenotypes. In control mice, Sulf1/2 and Slit2 proteins, produced by the radial glial cells, are present on the pial surface. In *Sulf1/2* DKO mice, increased 6-*O*-sulfated HS results in excessive accumulation of Slit2 protein (shown in dark blue) in the posterior hypothalamus, leading to dorsal displacement of the CST. Because DKO mice die within a day of birth, the CST caudal to the pyramidal decussation was not examined (dashed lines). In *Slit2* KO mice, the CST axons invade the ventral forebrain owing to the lack of repulsion by Slit2.

The elevated levels of Robo2-Fc binding in *Sulf1/2* DKO mice were restored to the control levels by electroporation of *Sulf1/2*, which was performed under the same conditions as those used for the phenotype rescue experiments, whereas electroporation of the mutant *Sulf1* was ineffective (Fig. 5c–e). The morphology of the basement membrane and radial glial cells, as assessed by laminin and nestin immunostaining, was normal in the *Sulf1/2* DKO mice (Supplementary Fig. S5f–i). In addition, no increase in *Slit2* mRNA expression was observed in the *Sulf1/2* DKO mice (data not shown). Taken together, these data indicate that abnormally high amounts of Slit2 protein accumulated in the ventral brain region, causing CST axon guidance defects in *Sulf1/2* DKO mice (Fig. 6).

### Abnormal Slit2 Protein Accumulation is Responsible for the CST Defects in *Sulf1/2* DKO Mice

Finally, we tested whether reduction of Slit2 levels in *Sulf1/2* DKO mice could restore the CST defects. First, we electroporated *Slit2* siRNA into DKO brains but failed to reduce the Slit2 levels significantly and the DKO phenotype was not rescued (data not shown). Next, we produced *Sulf1/2* DKO mice with heterozygous deletion of *Slit2* and found that the CST defects were not restored to normal. This was consistent with the finding that Robo2-Fc binding signals were still detected after deleting one allele of *Slit2* (Supplementary Fig. S5b,e). Finally, we tried to reduce Slit activity by means of Robo2-Fc overexpression, which was shown to be effective in neutralizing Slit activity *in vitro* ^25,26^. Strong overexpression of Robo2-Fc by pCX-*Robo2-Fc* electroporation restored the DKO phenotype on the electroporated side (n = 2/3 in *Sulf1/2* DKO mice; n = 3/6 in *Sulf1* ^−/−^;*Sulf2* ^−/−^;*Slit2* ^+/−^ mice; Fig. 5l–m). However, even in the rescued mice, the restoration was not complete: the CST axons turned medially towards the pons but were located laterally to the medial lemniscus, resembling the weak phenotype of *Sulf1/2* DKO mice. The incidence of phenotype recovery in pCX-*Robo2-Fc*-electroporated embryos was significantly higher than the appearance frequency of the weak phenotype in nonelectroporated *Sulf1/2* DKO mice (5/9 and 7/70 for electroporated and nonelectroporated mice, respectively; *P* = 0.003, Fisher exact test). These data indicate that suppression of Slit activity by Robo2-Fc overexpression led to the recovery of the CST defects in *Sulf1/2* DKO mice. The observation that Robo2-Fc did not completely rescue the CST defects might be explained by the weaker effects of other axon guidance molecules (for example, Sema3E). Taken together, our results demonstrate that abnormal accumulation of Slit2 protein is mainly responsible for the CST axon guidance defects in *Sulf1/2* DKO mice.

## Discussion

In this study, we have demonstrated that Sulf-mediated trimming of 6-*O*-sulfate groups in HS is required for CST axon guidance. Normally, Sulfs produced by radial glial cells modify HS sulfation patterns in the pial basement membrane. Loss of *Sulfs* results in increases in 6-*O*-sulfated HS, leading to accumulation of Slit2 protein and dorsal displacement of the CST. This contrasts sharply with the phenotype of *Slit/Robo* KO mice, in which CST axons invade the ventral brain owing to the lack of repulsion^21,27^ (Fig. 6). We propose a novel regulatory mechanism by which HS desulfation secures accurate axon guidance by tempering Slit2 protein accumulation. Although we have presented unequivocal evidence that Slit2 is a major cause for this particular CST phenotype, possible involvement of other molecules in other axon guidance defects is worthy of investigation in future.

Genetic studies in mice and zebrafish demonstrated requirement of HS biosynthesis in axon guidance^2,4,28–30^. More specifically, HS modification by specific sulfotransferases has also been implicated in axon guidance^2,4,31–36^. These studies indicate that presence of HS and its sulfate groups is essential for mediating axon guidance signals. Given that sulfate groups at specific positions in HS are required for the interaction between HS and signaling molecules^23^, desulfation is thought to be of the same importance as sulfation in the regulation of HS functions. Correspondingly, our present study has demonstrated that shaping of HS by removing sulfate groups at specific positions is important in axon guidance. Sulfs thus can act as a dynamic regulator that controls HS-dependent signaling in a spatiotemporal manner.

Although many lines of evidence indicate HS dependence of Slit/Robo signaling, how HS interacts with Slit/Robo and regulates their signaling *in vivo* remains largely elusive, particularly in the vertebrate brain. We devised a method to detect Slit2 protein on brain sections and clearly demonstrated the localization of Slit2 protein on the pial basement membrane in the *Sulf1/2* DKO brain. Moreover, we showed that Slit2 accumulation was associated with trisulfated HS disaccharides. Given that Slit2 binds strongly to 6-*O*-sulfated heparin^23^, it is likely that Slit2 accumulation in the DKO brain was due to increased stability of Slit2 by 6-*O*-sulfated HS. Interestingly, Robo2-Fc binding in the control brain was below the detection level in the examined area, suggesting rapid diffusion and/or degradation of Slit2 protein *in vivo*. This means that dissociation of Slit2 from HS by means of desulfation contributes to fine-tuning of Slit2 activity. In this context, it is noteworthy that in our proteomic analysis of the control brain, a Slit2 fragment was detected in a small-sized fraction (53–65 kDa), but not in the large-sized fraction corresponding to an active N-terminal Slit2 fragment (100–140 kDa).

Precise patterning of Slit localization is indispensable for accurate guidance of growing axons. In addition to HS, glycosylated dystroglycan and type IV collagen were implicated in the interaction with Slit protein and Slit-mediated axon guidance^37,38^. In particular, in zebrafish, Slit1 protein was shown to be translocated to the surface of the tectum by the actions of the radial glial cells and anchored to the basement membrane via interaction with type IV collagen^37^. Combined with these data, our findings suggest that transport of Slit protein to the brain surface by radial glial cells is a generalized mechanism controlling axon guidance. Two recent papers reported a similar mechanism in which netrin-1 protein produced by ventricular zone progenitor cells is transported to the pial surface to guide commissural axons in the spinal cord and hindbrain^39,40^, although the involvement of HS remains to be examined. Future studies are required to clarify how Sulfs play cooperative roles in anchoring guidance proteins in the basement membrane with dystroglycan and type IV collagen.

Our results also have an important implication in that Slit2 activity can be regulated by changing the HS microstructure in the extracellular environment without altering *Slit2* expression. Manipulation of the localization and activity of axon guidance molecules by Sulfs or heparin-related chemicals^41^ may be useful to control the action of chemorepellents for promoting regeneration of CST axons.

## Methods

### Knockout Mice

*Sulf* knockout mice were generated as described previously^19^. Offspring of mice backcrossed to C57BL/6N for 5 generations (N5 generation) were used. *Slit2* knockout mice were obtained from Mutant Mouse Regional Resource Centers. Noon of the day on which a vaginal plug was observed was taken as embryonic day 0.5 (E0.5). All animal experiments were approved by and performed according to the guidelines of the Animal Care and Use Committee of the University of Tsukuba.

### Disaccharide Analysis of Heparan Sulfate and Chondroitin Sulfate

Disaccharide analysis was performed as described previously^19^ by using neonatal brains because most of the DKO mice died within a day of birth. In brief, acetone-extracted neonatal mouse brains were treated with 0.8 mg/ml protease (P5147; Sigma) at 55 °C overnight, and then with 125 U benzonase (Sigma) at 37 °C for 2 h. After centrifugation, supernatants were purified with Vivapure D Mini M (Vivascience) and concentrated with Ultrafree-MC Biomax-5 (Millipore). The purified glycosaminoglycans were treated at 37 °C overnight with a mixture of 1 mIU of heparinase I (Sigma), 1 mIU of heparitinase I (Seikagaku), and 1 mIU of heparitinase II (Seikagaku) or with a mixture of 50 mIU of chondroitinase ABC (Seikagaku) and 50 mIU of chondroitinase ACII (Seikagaku). Unsaturated disaccharides were analyzed by ion-pair reversed-phase chromatography. An NaCl gradient (2–106 mM) in 1.2 mM tetra-*n*-butylammonium hydrogen sulfate (Wako Pure Chemical Industries) and 8.5% acetonitrile (Sigma) was applied on a Senshu Pak Docosil column (4.6 × 150 mm, particle size 5 μm; Senshu Scientific) at 55 °C using an HPLC system, the Alliance 2695 separations module (Waters Corporation). Postcolumn derivatization with 2-cyanoacetamide (Wako Pure Chemical Industries) was performed at 125 °C using a Post Column Reaction Module (Waters Corporation) and Temperature Control Module II (Waters Corporation). The effluent was monitored fluorometrically using a 2475 Multi-channel Fluorescence Detector (excitation 346 nm, emission 410 nm; Waters Corporation). Peaks were identified and quantified by comparison with authentic unsaturated disaccharide markers, an Unsaturated HS/HEP-Disaccharide Kit (H Mix; Seikagaku), ΔUA2S-GlcNAc, and ΔUA2S-GlcNAc6S (Dextra Laboratories), and an Unsaturated Chondro-Disaccharide Kit (C-Kit) (Seikagaku). The chromatogram was analyzed using Empower software (Waters Corporation), and the results were statistically analyzed using two-way ANOVA with a Bonferroni post hoc test.

### Histology and Immunohistochemistry

Cryostat (10-μm thick) sections of paraformaldehyde (PFA)-fixed brains were used for immunohistochemistry. To detect Sulf or Slit proteins and HS, fresh-frozen sections fixed with 95% ethanol and 1% acetic acid were used^42^. The primary antibodies used were anti-neurofilament-M (1:1000; Zymed), anti-FLAG (1:500; Abcam, ab6711), anti-GFP (1:1000; Molecular Probes), anti-Sulf1 (1:50; Abcam, ab32763), anti-Sulf2 (1:50; Santa Cruz, M79), anti-HS (1:5; AO4B08; refs^18,19^), anti-nestin (1:1000; Chemicon), and anti-laminin (1:1000; Sigma). The secondary antibodies used were peroxidase-conjugated anti-mouse IgG (1:200; Chemicon) and Alexa488- or Alexa568-conjugated anti-rabbit IgG (1:250; Molecular Probes). A chromogenic reaction was carried out using a VECTASTAIN Elite ABC kit and a DAB substrate kit (Vector Laboratories). For immunofluorescence, cell nuclei were stained with 3.3 μM TO-PRO-3 iodide (Molecular Probes). Whole-mount immunostaining was done (with minor modifications) using the same reagents after the meninges were removed^43^.

### DiI Tracing of the Corticospinal Tract

DiI (1,1′-dioctadecyl-3,3,3′,3′-tetramethylindocarbocyanine perchlorate; Molecular Probes), dissolved in dimethylformamide, was injected into the motor cortices of neonatal mice by the use of glass micropipettes. The mice were perfusion-fixed with 4% PFA/PBS approximately 10 h after the injection. The dissected brains and vibratome sections (100-μm thick) were observed and photographed using a fluorescence stereoscopic microscope (MZ FL III; Leica) and a fluorescence microscope (Axioplan2; Zeiss), respectively.

### *In Situ* Hybridization

*In situ* hybridization was performed as described previously^44^. In brief, 10-μm-thick cryostat sections were hybridized with a 1 μg/ml digoxigenin (DIG)-labeled antisense RNA probe at 65 °C for 16 h. After washing, the slides were incubated with an alkaline phosphatase-conjugated anti-DIG antibody (Roche) at 4 °C for 16 h. Signals were detected using BM purple (Roche Diagnostics) in the presence of 2 mM levamisole (Sigma) at room temperature for 3 to 5 d.

### *In Utero* Electroporation

*In utero* electroporation was performed as previously described^44^. The cDNAs were subcloned into a pCX or pEF-BOS vector^16,17^. A DNA solution (2.0–2.5 μl) containing a total of 600 nM of the expression constructs was injected into the lateral ventricle of E12.5 or E13.0 mouse embryos. Five square electric pulses (35 V, 50 ms duration, 1 pulse/s) were delivered using an electroporator (CUY21; Nepa Gene) and a 3-mm electrode (CUY650P3; Nepa Gene).

### Plasmid Constructs for Electroporation

The plasmids used in this study were obtained as follows. EGFP was derived from pEGFP-N3 (Clontech). Rat *Sulf1* (*SulfFP1*) and *Sulf2* (*SulfFP2*) were previously described^19,45^. The point mutation in *Sulf1*(*C87A*) was introduced by PCR. Mouse cDNAs *Slit1* (BC062091), *Sema3e* (IMAGE:5357516), *Sema4d* (IMAGE:6509473), *Sema4g* (IMAGE:6395010), *Sema5b* (IMAGE:5719939), and *Efnb2* (IMAGE:6827408) were obtained from the IMAGE consortium clone collection. Mouse *Slit2* (G730002D07) and *Sema6b* (G730036J03) were obtained from RIKEN Mouse FANTOM Clones (DNAFORM). Because both the *Slit1* and the *Slit2* clones contained short deletions, the incorrect sequences were replaced with the correct sequences that were amplified by RT-PCR, followed by sequence confirmation. Rat *Robo1* and *Robo2* cDNAs were amplified by RT-PCR, and their whole sequences determined to confirm that they contained the correct sequences.

Sulf1 was tagged with either a FLAG peptide (DYKDDDDK) or a Myc peptide (EQKLISEEDL) plus the 6xHis tag at its C-terminus. Sulf2 was tagged with the Myc peptide at its C-terminus. *Sulf* cDNAs were subcloned into the pCX vector^16^ and pEF-BOS vector^17^. For the rescue experiments, pCX-*Sulf1*-FLAG, pCX-*Sulf2*-Myc, pEF-BOS-*Sulf1*-FLAG, and pEF-BOS-*Sulf2*-Myc were used; for the sake of simplicity, they are denoted in the text as pCX-*Sulf1*, pCX-*Sulf2*, pEF-BOS-*Sulf1*, and pEF-BOS-*Sulf2*, respectively. In the experiments in which Sulf protein localization was examined, *Sulf1*-Myc-His and *Sulf2*-Myc were subcloned into a pCX vector containing a Kozak sequence (GCCACCATG; the underline is the initiation codon), a signal sequence from the human *CD8A* gene (ALPVTALLLPLALLLHAARP), the FLAG peptide, and the 6xHis tag. Sulf1 and Sulf2 are connected to the above sequence from the positions of the 20th and 25th amino acids, respectively (both are the N-termini of the mature protein predicted by the SignalP 3.0 algorithm [http://www.cbs.dtu.dk/services/SignalP/]). For simplicity, the expression constructs created, pCX-Kozak-SS-FLAG-His-*Sulf1*-Myc-His and pCX-Kozak-SS-FLAG-His-*Sulf2*-Myc, are denoted in the text as pCX-FLAG-*Sulf1* and pCX-FLAG-*Sulf2*, respectively.

The mouse cDNAs *Slit1*, *Slit2*, *Sema3e*, *Sema4d*, *Sema4g*, *Sema5b*, *Sema6b*, and *Efnb2* were subcloned into a pCX vector (and a pEF-BOS vector for *Slits*) that contained the Kozak sequence, the signal sequence from the human *CD8A* gene, the FLAG peptide, and the 6xHis tag. Slit1, Slit2, Sema3E, Sema4D, Sema4G, Sema5B, Sema6B, and EphrinB2 were connected to the above sequence from the positions of the 33^rd^, 26^th^, 26^th^, 24^th^, 18^th^, 27^th^, 17^th^, and 30^th^ amino acids, respectively (all are the N-termini of the mature protein predicted by the SignalP 3.0 algorithm). For simplicity, they are denoted in the text as pCX-*Slit1*, pCX-*Slit2*, pCX-*Sema3e*, pCX-*Sema4d*, pCX-*Sema4g*, pCX-*Sema5b*, pCX-*Sema6b*, and pCX-*Efnb2*, respectively. In the *in vitro* Robo2-Fc binding experiments, rat Slit2 tagged with an HA sequence (YPYDVPDYA) was subcloned into a pCEP4 vector (Invitrogen) containing the Kozak sequence, the signal sequence from the human *CD8A* gene, the FLAG peptide, and the 6xHis tag.

### LC-MS/MS Analysis

Liquid choromatography-ion trap mass spectrometry (LC-MS/MS) analysis was performed as described previously^46^. Briefly, the samples were separated by SDS-PAGE and the gel was cut into 15 pieces. The gel pieces were repeatedly soaked in 25 mM triethylammonium bicarbonate (TEAB), pH 8.0, containing 50% acetonitrile for 30 min. After being dried in a Savant Speed-Vac concentrator (Thermo Fisher Scientific), the gel was incubated in 25 mM TEAB, pH 8.0, containing 75–150 ng of modified trypsin (Roche Diagnostics) at 37 °C for 16–20 h. The digest were extracted twice with 100–300 μl of 0.1% trifluoroacetic acid containing 60% acetonitrile. These 2 extracts were combined, dried in a Speed-Vac concentrator, and kept at −80 °C until the assay. The sample was resuspended in 0.1% formic acid containing 2% acetonitrile and applied to a DiNa HPLC system (KYA Technologies Corporation). A reverse-phase capillary column (Develosil ODS-HG5, 0.075 mm i.d. × 150 mm; Nomura Chemical) was used at a flow rate of 200 or 300 nl/min with a 4–80% linear gradient of acetonitrile. Eluted peptides were directly detected with an ion trap mass spectrometer (LXQ; Thermo Fisher Scientific) at a spray voltage of 1.9 kV and collision energy of 35%. The mass acquisition method consisted of 1 full MS survey scan followed by MS/MS scans of the most abundant precursor ions from the survey scan. Dynamic exclusion for the MS/MS spectra was set to 30 s. The data were analyzed with BioWorks (Thermo Fisher Scientific) and Mascot (Matrix Science) software.

### Robo2-Fc Binding Assay

Cos-7 cells were transfected with pMT21-Robo2-Fc using LipofectAMINE Plus reagent (Life Technologies) or FuGene HD (Promega). After the cells were cultured in serum-free Opti-MEM I (Life Technologies) for 3 days, the conditioned medium was concentrated about 5-fold using an Amicon Ultra-15 filter (50k MCO; Millipore). For *in vitro* assays, 293EBNA cells (Life Technologies) transfected with pCEP4-FLAG-*Slit1* or pCEP4-FLAG-*Slit2* were incubated with Robo2-Fc in a PH buffer (PBS with 1% heat-inactivated normal goat serum) at room temperature for 1 h. After being washed, the cells were fixed with 2% PFA/PBS, permeabilized in PHT (PH with 0.1% Triton-X100), and incubated with Cy3-labeled anti-human IgG antibody at room temperature for 30 min (1:350; Jackson ImmunoResearch).

For *in situ* detection, 10-μm-thick fresh-frozen sections of E15.5 mouse heads were fixed in 1% acetic acid and 95% ethanol at −20 °C overnight. After being washed with PBS and blocked with PHT at room temperature for 1 h, the slides were incubated with Robo2-Fc (about 2.5-fold concentrated) at 4 °C overnight. The slides were then washed with PHT and incubated with biotin-SP-conjugated anti-human IgG antibody (1:50; Jackson ImmunoResearch) at room temperature for 45 min and then with Alexa546-conjugated streptavidin (1:50; Life Technologies) at room temperature for 15 min. In this preparation, the fluorescence signal of elecroporated EGFP was eliminated, and thereby the immunostaining was not disturbed.

A series of z-stack fluorescence images (1024 × 1024 pixels) was obtained using a laser-scanning microscope, the LSM510 with a 10x objective lens (Carl Zeiss). For quantitative comparison of the fluorescence intensity of the samples, the same parameters (pinhole, 80.3 μm; optimal interval, 6.54 μm; detector gain, 1000; amplifier offset, −0.1; amplifier gain, 1; scan speed, 9; average number, 2) were used for all scans. Three sections were used for each experimental condition. In each section, the single optical slice with the strongest intensity was selected for measurement. An outline of the brain surface was traced, and the fluorescence intensity in the traced area was measured.

### Western Blot Analysis

Meninges or brains dissected from the hypothalamic and midbrain regions of E15.5 mouse embryos were homogenized in a sample buffer (62.5 mM Tris-HCl, pH 6.8, 2% SDS, 5% sucrose, 0.01% bromophenol blue, 10% 2-mercaptoethanol). The primary antibodies used were anti-Slit2 (1:200, G-19; Santa Cruz Biotechnology), anti-actin (1:1000; Sigma), and anti-laminin (1:1000; Sigma). The secondary antibodies used were peroxidase-conjugated anti-goat or anti-rabbit IgG (1:2500; Jackson ImmunoResearch). Signals were detected using the ECL Plus Western Blotting Detection System (GE). The data of a control experiment that confirmed the specificy of anti-Slit2 antibody (G-19) are shown in Supplemtary Fig. S6.

### Statistics

No statistical methods were used to predetermine sample sizes, but our sample sizes are similar to those generally employed in the field. Statistical analyses for the data of Robo2-Fc binding and disaccharide analysis were done with ANOVA with Tukey-Kramer and Bonferroni post hoc tests, respectively. The Fisher exact test was used to analyze the incidence of phenotype recovery in pCX-*Robo2-Fc*-electroporated embryos. Welch’s t-test and a paired t-test were used to analyze the average fluorescence signal intensity, the width of the axon bundles, and the area of the neurofilament positive area (details of the analysis are described in Supplementary Table 1).

### Data availability

The data that support the findings of this study are availiable from the corresponding author upon reasonable request.

## Electronic supplementary material

Supplementary information
