# Supplementary material for: Desulfation of Heparan Sulfate by Sulf1 and Sulf2 Is Required for Corticospinal Tract Formation
Source: Sci Rep. 2017 Oct 23;7:13847. doi: 10.1038/s41598-017-14185-3 (PMC5653861; doi:10.1038/s41598-017-14185-3)
Supplement: Supplementary file 1 — Supplementary information [file 41598_2017_14185_MOESM1_ESM.pdf]

Supplementary Information

Desulfation of Heparan Sulfate by Sulf1 and Sulf2 Is Required for Corticospinal Tract Formation

Takuya Okada[1,+], Kazuko Keino-Masu[1,+], Satoshi Nagamine[1,6], Fuyuki Kametani[4], Tatsuyuki Ohto[1,2], Masato Hasegawa[4], Toin H. van Kuppevelt[5], Satoshi Kunita[3,7], Satoru Takahashi[3], Masayuki Masu[1]

[1]Department of Molecular Neurobiology, Faculty of Medicine,
[2]Department of Pediatrics, University of Tsukuba Hospital, and
[3]Laboratory Animal Resource Center,
University of Tsukuba, 1-1-1 Tennodai, Tsukuba, Ibaraki 305-8575, Japan
[4]Department of Neuropathology and Cell Biology, Tokyo Metropolitan Institute of Medical Science, Setagaya-ku, Tokyo 156-8506, Japan
[5]Department of Biochemistry, Nijmegen Institute for Molecular Life Sciences, Radboud University Medical Center, Nijmegen, the Netherlands

[6]Present address: Pharmaceuticals and Medical Devices Agency, 3-3-2 Kasumigaseki, Chiyoda-Ku, Tokyo 100-0013, Japan
[7]Present address: Center for Experimental Medicine, Jichi Medical University, 3311-1 Yakushiji, Shimotsuke, Tochigi 329-0498, Japan
[+]These authors contributed equally to this work.

Correspondence should be addressed to:
Masayuki Masu
Department of Molecular Neurobiology, Faculty of Medicine, University of Tsukuba, 1-1-1 Tennodai, Tsukuba, Ibaraki 305-8575, Japan
Phone: +81-29-853-3249
Fax: +81-29-853-3498
E-mail: mmasu@md.tsukuba.ac.jp

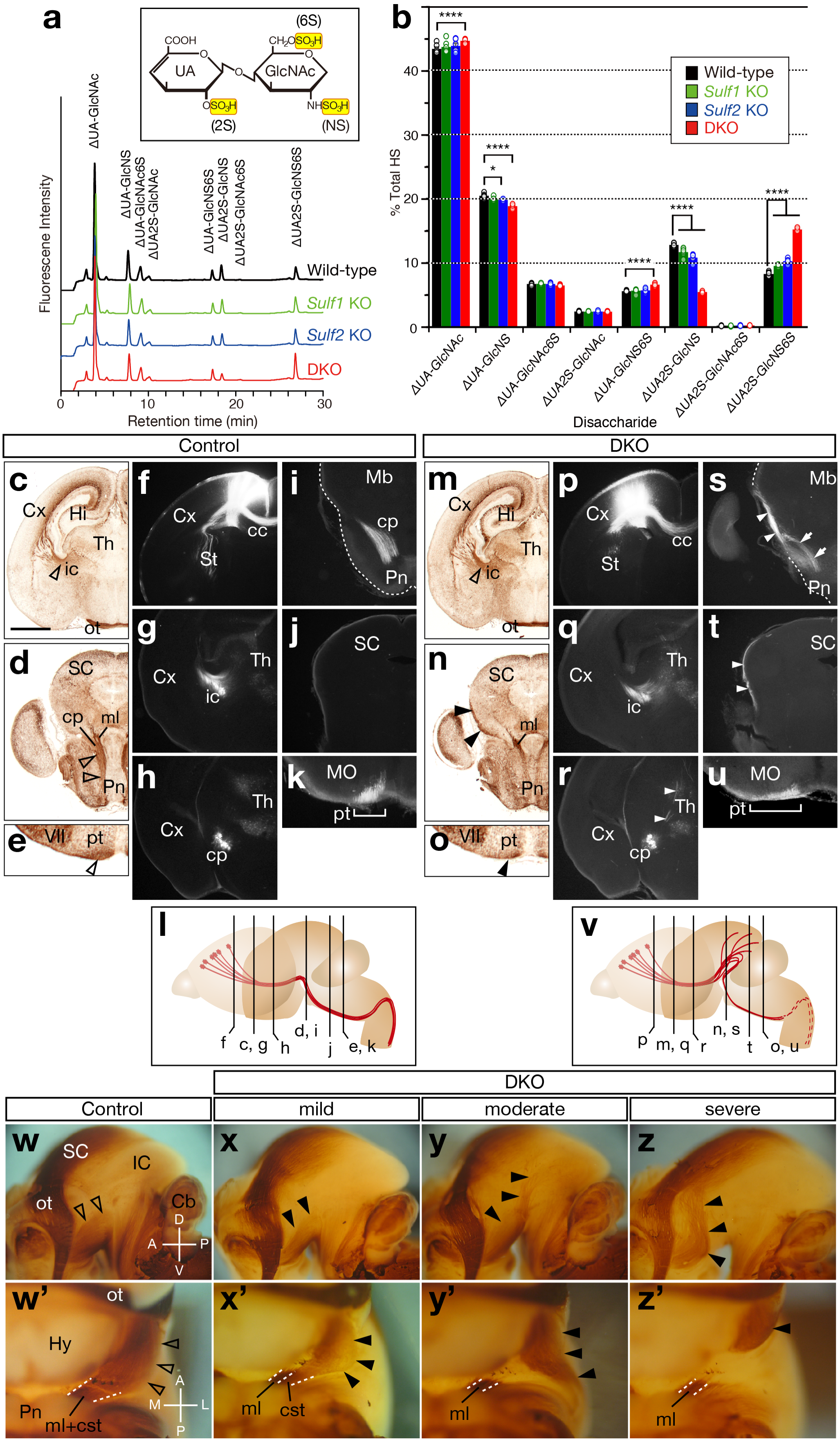
a
b
COOH
UA
GlcNAc
(6S)
CH_2OSO_3H
OSO_3H
(2S)
$NHSO_3H$
(NS)
Fluorescene Intensity
Retention time (min)
ΔUA-GlcNAc
ΔUA-GlcNS
ΔUA-GlcNAc6S
ΔUA2S-GlcNAc
ΔUA-GlcNS6S
ΔUA2S-GlcNS
ΔUA2S-GlcNAc6S
ΔUA2S-GlcNS6S
Wild-type
Sulf1 KO
Sulf2 KO
DKO
% Total HS
Disaccharide
Control
DKO
Cx
Hi
Th
ic
ot
SC
cp
ml
Pn
VII
pt
St
cc
Mb
MO
d, i
c, g
e, k
n, s
m, q
o, u
mild
moderate
severe
IC
Cb
Hy
ml+cst
cst

Supplementary Figure S1. HS Disaccharide Profile and CST Defects in *Sulf1/2* DKO Mice.

(**a**) HPLC chromatograms showing HS disaccharide profiles of neonatal brains. Unsaturated HS disaccharides obtained by heparin lyase digestion were analyzed using ion-pair reversed-phase chromatography with postcolumn derivatization. The inset shows the structure of an HS disaccharide.

(**b**) Molar percentages of 8 disaccharides in the HS from the neonatal brains of the indicated genotypes. Values are the means ± S.E.Ms. (n = 6, two-way ANOVA with a Bonferroni post hoc test). *Sulf1* KO and *Sulf2* KO brains showed increase in the trisulfated disaccharide ΔUA2S-GlcNS6S (wild-type, 8.3 ± 0.1% versus *Sulf1* KO and *Sulf2* KO, 9.5 ± 0.1% and 10.3 ± 0.1%, respectively; 6 mice; ****, $P < 0.0001$, two-way ANOVA with Bonferroni post hoc test) and decrease in the disulfated disaccharide ΔUA2S-GlcNS (wild-type, 12.8 ± 0.1% versus *Sulf1* KO and *Sulf2* KO, 11.7 ± 0.3% and 10.8 ± 0.2%, respectively; ****, $P < 0.0001$). In *Sulf1/2* DKO brains, the changes were more than a simple additive effect of the 2 single mutants (ΔUA2S-GlcNS6S, 15.2 ± 0.1%; ΔUA2S-GlcNS, 5.5 ± 0.04%; ****, $P < 0.0001$ versus wild-type), indicating genetic redundancy of *Sulf1* and *Sulf2*. In addition, *Sulf1/2* DKO brains showed increase in ΔUA-GlcNS6S (wild-type, 5.6 ± 0.04% versus *Sulf1/2* DKO, 6.6 ± 0.1%; ****, $P < 0.0001$) and decrease in ΔUA-GlcNS (wild-type, 20.5 ± 0.1% versus DKO, 18.8 ± 0.1%; ****, $P < 0.0001$), indicating that Sulfs have weak 6-*O*-desulfation activity toward UA-GlcNS6S units in HS. The sulfation profiles of chondroitin sulfate were not changed in the mutant brains (data not shown).

(**c**-**e**, **m**-**o**) Immunohistochemistry for neurofilament-M in coronal sections of E18.5 brains of control (*Sulf1*$^{-/-}$) and *Sulf1/2* DKO mice. Closed arrowheads in (**n**, **o**) indicate abnormal fibers on the midbrain surface and reduced sizes of the pyramidal tracts (pt). Open arrowheads in (**c**-**e**) indicate normal CST in the control mice.

(**f**-**k**, **p**-**u**) Coronal sections of the DiI-injected brains. In *Sulf1/2* DKO mice, aberrant subcortical projections towards the tectum through the thalamus (arrowheads, **r**) were observed. In the midbrain, abnormal axons extended onto the lateral surface of the brain (arrowheads, **s**, **t**). Most abnormal axons returned to the pons but were positioned more laterally (arrows, **s**). In the medulla, the pyramidal tract became thin and broad (**u**).

(**l**, **v**) Trajectory of the CST. Vertical lines indicate the positions of the sections in (**c**-**k**) and (**m**-**u**).

(**w**-**z**, **w'**-**z'**) Whole-mount neurofilament-M immunostaining of E18.5 brains. Lateral (**w**-**z**) and ventral views (**w'**-**z'**) of the control (**w**, *Sulf1*$^{+/-}$;*Sulf2*$^{-/-}$) and DKO (**x**-**z**) brains are shown. Cerebral cortices were removed to make the midbrain visible. Open and closed arrowheads indicate normal and abnormal CST axons, respectively. In a mild case, the CST turns medially towards the pons and runs immediately lateral to the medial lemniscus (ml). In a severe case, the CST projects to the superior colliculus and does not extend towards the pons.

VII, facial nucleus; cp, cerebral peduncle; Cb, cerebellum; cc, corpus callosum; Cx, cerebral cortex; Hi, hippocampus; Hy, hypothalamus; IC, inferior colliculus; ic, internal capsule; ot, optic tract; Pn, pons; SC, superior colliculus; St, striatum; Th, thalamus. Anterior-posterior (A-P), dorsal-ventral (D-V), and medial-lateral (M-L) body axes are shown. Scale bars indicate 800 μm (**c**, **d**, **m**, **n**), 350 μm (**e**, **k**, **o**, **u**), 750 μm (**f**-**j**, **p**-**t**), 650 μm (**w**-**z**), and 350 μm (**w'**-**z'**).

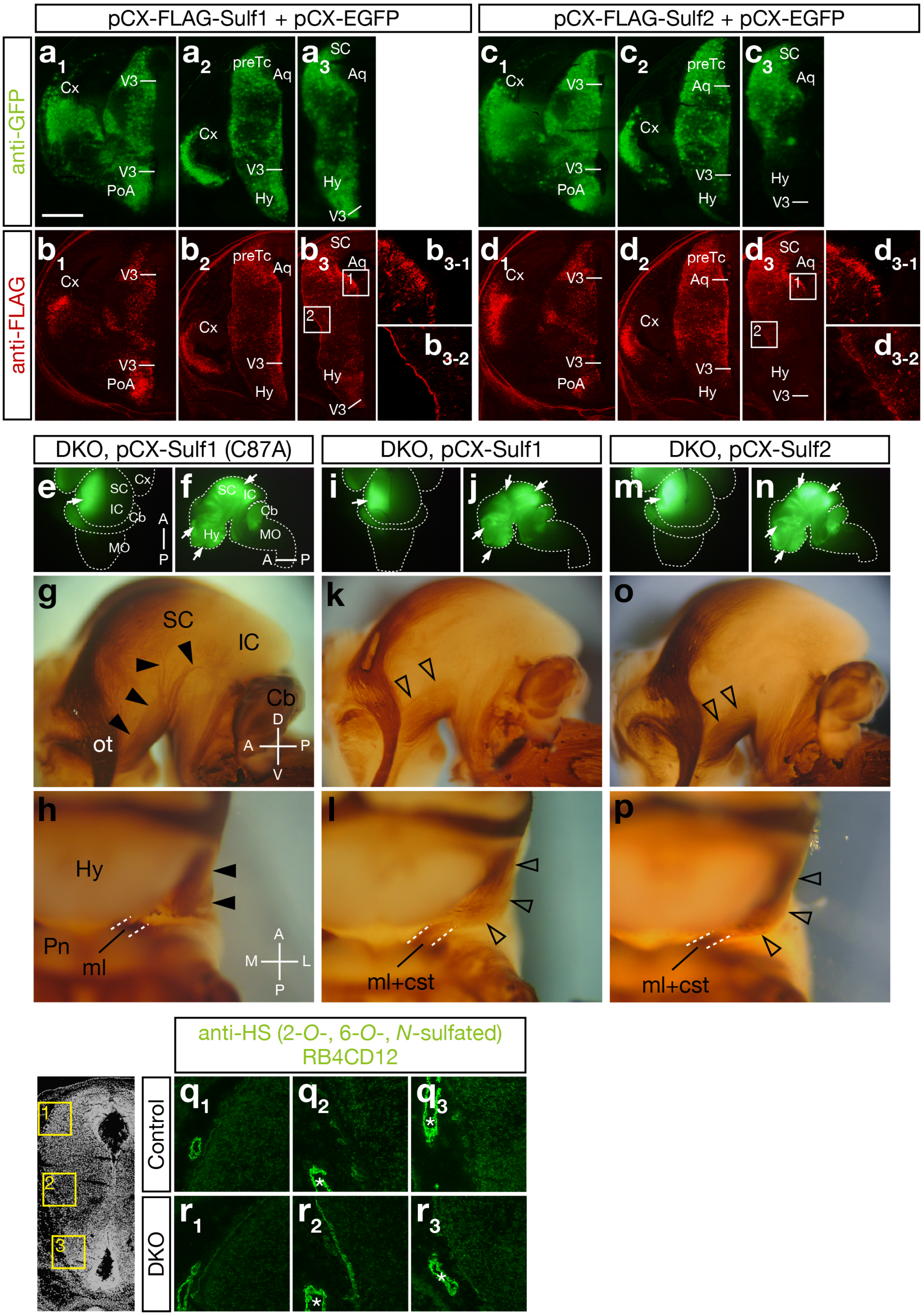
pCX-FLAG-Sulf1 + pCX-EGFP
pCX-FLAG-Sulf2 + pCX-EGFP
anti-GFP
anti-FLAG
Cx
V3
PoA
preTc
Aq
Hy
SC
DKO, pCX-Sulf1 (C87A)
DKO, pCX-Sulf1
DKO, pCX-Sulf2
IC
Cb
MO
ot
Pn
ml
ml+cst
anti-HS (2-O-, 6-O-, N-sulfated)
RB4CD12
Control
DKO

Supplementary Figure S2. Localization of Exogenous Sulf Proteins and *In Vivo* Rescue of the CST Defects in *Sulf1/2* DKO Mice.

(**a**-**d**) Colocalization of EGFP and Sulf proteins in coelectroporation. Wild-type brains were electroporated with either pCX-FLAG-*Sulf1* or pCX-FLAG-*Sulf2* together with pCX-*EGFP* at E12.5, and protein localization was examined at E15.5. The fresh-frozen sections were alternately fixed with 4% PFA or ethanol/acetate, and then stained with anti-GFP antibody in the PFA-fixed sections (**a**, **c**) and with anti-FLAG antibody in the ethanol/acetate-fixed sections (**b**, **d**). Outermost signals in anti-FLAG staining are the nonspecific ones in the skull and skin (**b**, **d**). The pictures ($\mathbf{b_{3\text{-}1}}$) and ($\mathbf{d_{3\text{-}1}}$) show the magnified images of the ventricular zone in the boxed areas in ($\mathbf{b_3}$) and ($\mathbf{d_3}$), respectively. The pictures ($\mathbf{b_{3\text{-}2}}$) and ($\mathbf{d_{3\text{-}2}}$) show the magnified images of the pial surface in the boxed areas in ($\mathbf{b_3}$) and ($\mathbf{d_3}$), respectively.

(**e**-**p**) Electroporation-mediated rescue of the CST defects in *Sulf1/2* DKO mice. The indicated plasmids together with pCX-*EGFP* were electroporated into E12.5 *Sulf1/2* DKO brains. At E18.5, EGFP expression and the CST trajectory were examined. Electroporation of *Sulf1* or *Sulf2* into the region from the hypothalamus to the midbrain could rescue the DKO phenotype (**i**-**p**), whereas a mutant Sulf1 lacking enzyme activity, in which a critical cysteine residue at 87 was replaced by alanine, could not rescue the phenotype (**e**-**h**). Arrows indicate the sites of electroporation. Anterior-posterior (A-P), dorsal-ventral (D-V), and medial-lateral (M-L) body axes are shown. Statistical analyses of the neurofilament-positive fibers in the midbrain (**g**, **k**, **o**) are shown in **Supplementary Table 1**.

(**q**, **r**) Immunohistochemistry of anti-HS RB4CD12 antibody in the control brain ($\mathbf{q_1}$-$\mathbf{_3}$) and *Sulf1/2* DKO brain ($\mathbf{r_1}$-$\mathbf{_3}$). The pictures ($\mathbf{q_1}$-$\mathbf{r_1}$), ($\mathbf{q_2}$-$\mathbf{r_2}$), and ($\mathbf{q_3}$-$\mathbf{r_3}$) show the boxed areas (numbered 1-3) in the left panel. Asterisks show blood vessels. The signals were strongly detected in the cerebral peduncle of the *Sulf1/2* DKO brain.

Aq, aqueduct; Cb, cerebellum; cst, corticospinal tract; Cx, cerebral cortex; Hy, hypothalamus; IC, inferior colliculus; ml, medial lemniscus; MO, medulla oblongata; ot, optic tract; PoA, preoptic area; preTc, pretectum; SC, superior colliculus; V3, third ventricle.

Scale bars indicate 725 μm (**a**-**d**), 250 μm ($\mathbf{b_{3\text{-}1}}$, $\mathbf{b_{3\text{-}2}}$, $\mathbf{d_{3\text{-}1}}$, $\mathbf{d_{3\text{-}2}}$), 2.0 mm (**e**, **f**, **i**, **j**, **m**, **n**), 650 μm (**g**, **k**, **o**), 350 μm (**h**, **l**, **p**), and 200 μm (**q**, **r**).

a

Meninges			
Fraction	Size (kDa)	Control	DKO
1	<10		
2	10-19		
3	19-25		
4	25-30		
5	30-45	Efnb2	
6	45-55		
7	55-65		
8	65-90		Sema3e
		Sema4d	
			Sema5b
			Sema6b
9	90-110		Sema4g
10	110–150	Slit1	Slit1
			Slit2
11	150-185		
12	185-250		
13-15	>250		

Brain parenchyme + Meninges			
Fraction	Size (kDa)	Control	DKO
1	<10		
2	10-15		
3	15-23		Sema4c
4	23-30		
5	30-35		Epha4
6	35-42	Slitrk6	Slitrk6
7	42-53		
8	53-65	Slit2	
9	65-80	Sema4d	
10	80-100	Netrin-G1	Netrin-G1
		Epha5	Epha5
		Ephb1	Ephb1
		Ephb2	Ephb2
11	100-140		Slit2
		Epha2	Epha2
		Epha3	Epha3
		Epha4	Epha4
		Epha5	Epha5
		Epha6	
		Epha7	
		Epha8	
		Ephb1	Ephb1
		Ephb2	Ephb2
		Ephb3	
		Ephb4	
12	140-185	Slit1	Slit1
		Ephb1	Ephb1
		Ephb2	Ephb2
13	180-250		Ephb6
14-15	>250		Ephb2

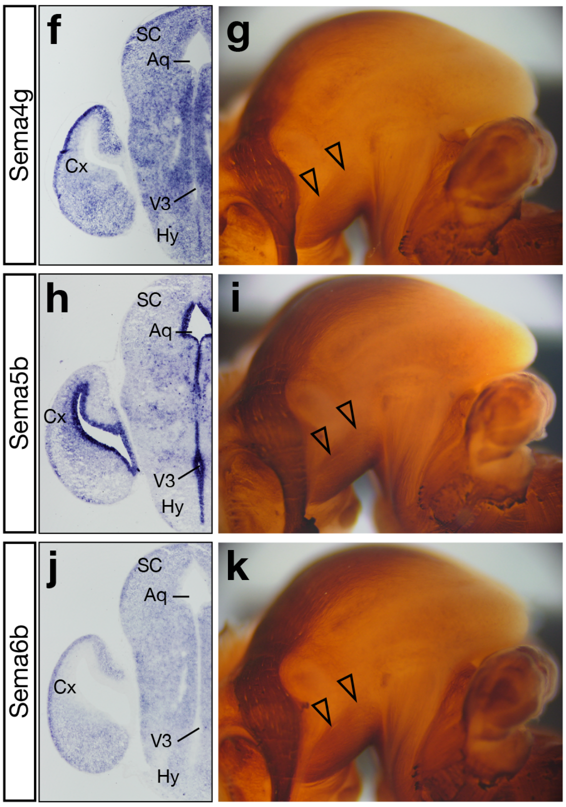

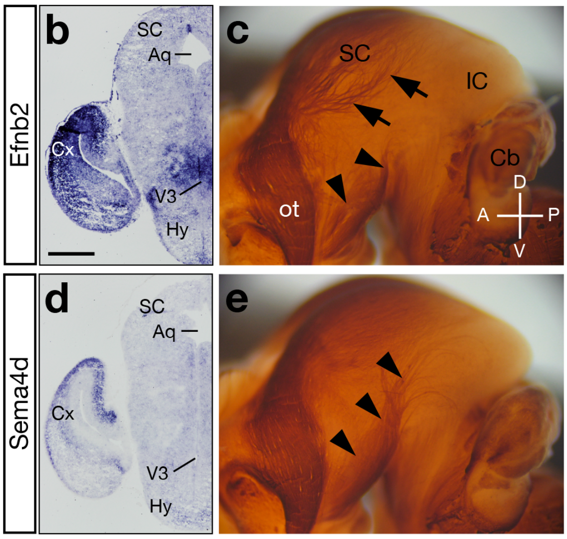

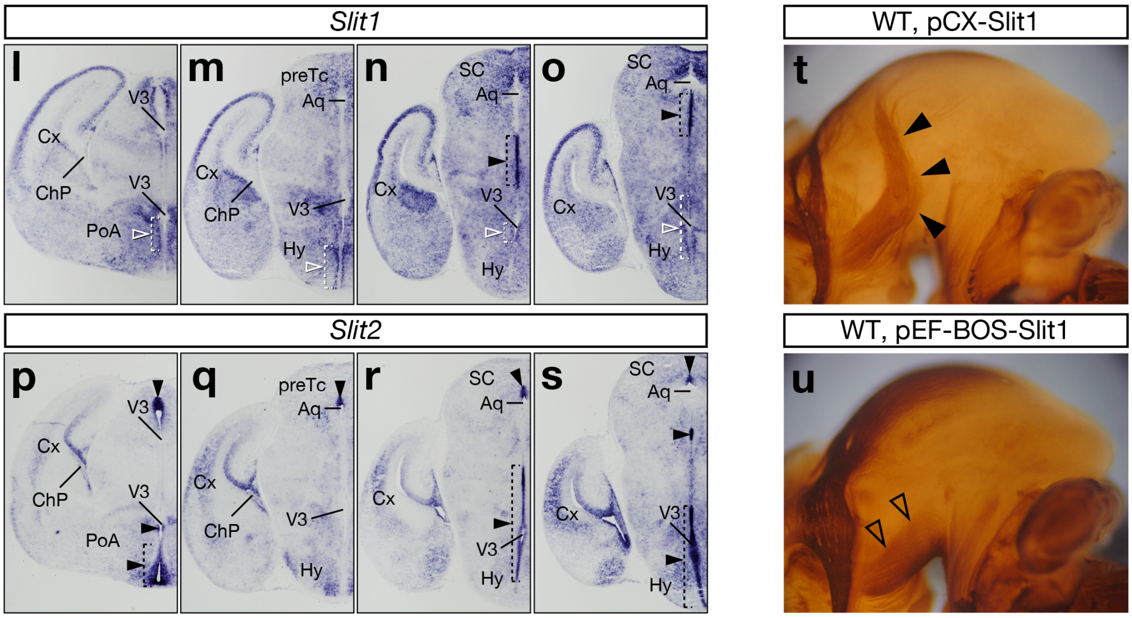

Supplementary Figure S3. Expression Patterns of EphrinB2, Semaphorins, and Slits, and Effects of Their Overexpression on the CST.

(**a**) Summary of proteomic analysis. Gene symbols are shown.

(**b**, **d**, **f**, **h**, **j**) *In situ* hybridization of the indicated genes in coronal sections of the E15.5 brain.

(**c**, **e**) Electroporation of *Efnb2* and *Sema4d* into *Sulf1/2* DKO brains at E12.5 did not rescue the DKO phenotype. Closed arrowheads in (**c**) and (**e**) indicate abnormal CST. Electroporation of *Efnb2* induced abnormal cell clustering (data not shown) and defects in retinotectal projection (**c**, arrows).

(**g**, **i**, **k**) Electroporation of *Sema4g*, *Sema5b*, and *Sema6b* into wild-type brains at E12.5 did not induce CST defects. Open arrowheads indicate normal CST.

(**l-s**) *In situ* hybridization of *Slit1* and *Slit2* in coronal sections of the E15.5 brain. Closed arrowheads indicate *Slit* mRNA expression in the ventricular zone of the third ventricle. *Slit1* mRNA was broadly expressed, including in the dorsal portion of the ventricular zone and the ventral portion of the subventricular zone (open arrowheads). *Slit2* mRNA was detected in the ventricular zone of the posterior hypothalamus.

(**t**, **u**) Effects of *Slit1* overexpression on CST axons. The indicated plasmids together with pCX-*EGFP* were electroporated into wild-type brains at E12.5 (**t**) or E13.0 (**u**). CST trajectories were examined at E18.5. Electroporation of pCX-*Slit1* induced CST defects (closed arrowheads) that were similar to those induced by *Slit2* overexpression, whereas electroporation of pEF-BOS-*Slit1* did not induce significant changes in the CST (open arrowheads).

Aq, aqueduct; Cb, cerebellum; ChP, choroid plexus; Cx, cerebral cortex; FM, foramen of Monro; Hy, hypothalamus; IC, inferior colliculus; MO, medulla oblongata; ot, optic tract; PoA, preoptic area; preTc, pretectum; SC, superior colliculus; Sp, septum; Th, thalamus; V3, third ventricle. Anterior-posterior (A-P) and dorsal-ventral (D-V) body axes are shown.

Scale bars indicate 550 μm (**b**, **d**, **f**, **h**, **j**, **l-s**) and 600 μm (**c**, **e**, **g**, **i**, **k**, **t**, **u**).

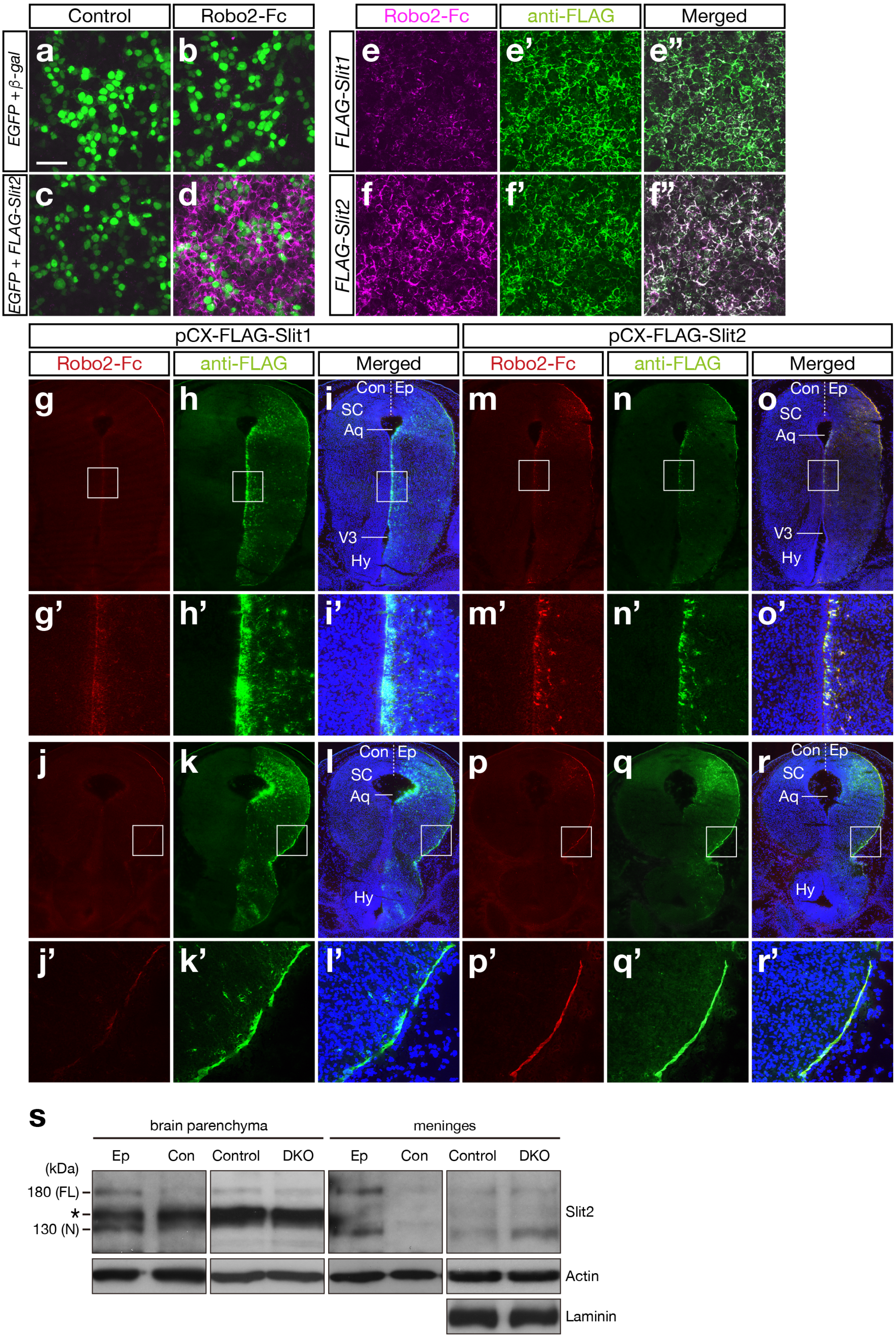
Control
Robo2-Fc
EGFP + β-gal
EGFP + FLAG-Slit2
a
b
c
d
Robo2-Fc
anti-FLAG
Merged
FLAG-Slit1
FLAG-Slit2
e
e'
e''
f
f'
f''
pCX-FLAG-Slit1
pCX-FLAG-Slit2
Robo2-Fc
anti-FLAG
Merged
Robo2-Fc
anti-FLAG
Merged
g
h
i
m
n
o
Con
Ep
SC
Aq
V3
Hy
g'
h'
i'
m'
n'
o'
j
k
l
p
q
r
j'
k'
l'
p'
q'
r'
s
brain parenchyma
meninges
Ep
Con
Control
DKO
Ep
Con
Control
DKO
(kDa)
180 (FL)
*
130 (N)
Slit2
Actin
Laminin

Supplementary Figure S4. Robo2-Fc Binding Assay and Western Blot Analysis.

(**a**-**d**) Robo2-Fc binding on Slit2-expressing cells. 293EBNA cells transfected with the indicated genes were incubated with conditioned media from mock-transfected cells (**a**, **c**) or Robo2-Fc-transfected cells (**b**, **d**). Robo2-Fc protein bound to the surface of the unpermeabilized cells was detected by Cy3-conjugated anti-human IgG antibody.

(**e**, **f**) Robo2-Fc binding on Slit1- or Slit2-expressing cells. Robo2-Fc binding was colocalized with anti-FLAG staining, indicating that Robo2-Fc protein bound to Slit1 and Slit2. Robo2-Fc binding appeared to be stronger for Slit2 than for Slit1.

(**g**-**r**) Robo2-Fc binding on pCX-FLAG-*Slit1*- or pCX-FLAG-*Slit2*-electroporated brain sections. Robo2-Fc binding was colocalized with anti-FLAG immunostaining. In the ventricular zone of the third ventricle, the signals were observed in the cell bodies (**g**-**i**, **m**-**o**). In the posterior hypothalamus and superior colliculus, Robo2-Fc binding and anti-FLAG staining were observed on the meninges (**j**-**l**, **p**-**r**). (**g'**)-(**r'**) show high-magnification pictures of the boxed areas in (**g**)-(**r**). Con, control side; Ep, electroporated side. Robo2-Fc binding appeared to be stronger for Slit2 than for Slit1. Aq, aqueduct; Hy, hypothalamus; SC, superior colliculus; V3, 3rd ventricle.

Scale bars: 50 μm (**a**-**f**), 500 μm (**g**-**r**), 100 μm (**g'**-**r'**).

(**s**) Western blot analysis of Slit2. Brain parenchyma and meninges dissected from the hypothalamic and midbrain regions of E15.5 mouse embryos were subjected to Western blot analysis. Wild-type brains were electroporated with pCX-*Slit2* at E12.5, and the control side (Con) and electroporated side (Ep) were compared. Nonelectroporated brains from control mice and *Sulf1/2* DKO mice were compared. Actin and laminin were the loading controls. Slit2 proteins (about 180 kDa and 130 kDa) were detected in the brain parenchyma and meninges of *Slit2*-electroporated brains. FL and N indicate the full-length and N-terminal fragments of Slit2, respectively. Slit2 was detectable in the meninges of nonelectroporated control and DKO mice. The asterisk indicates a nonspecific band.

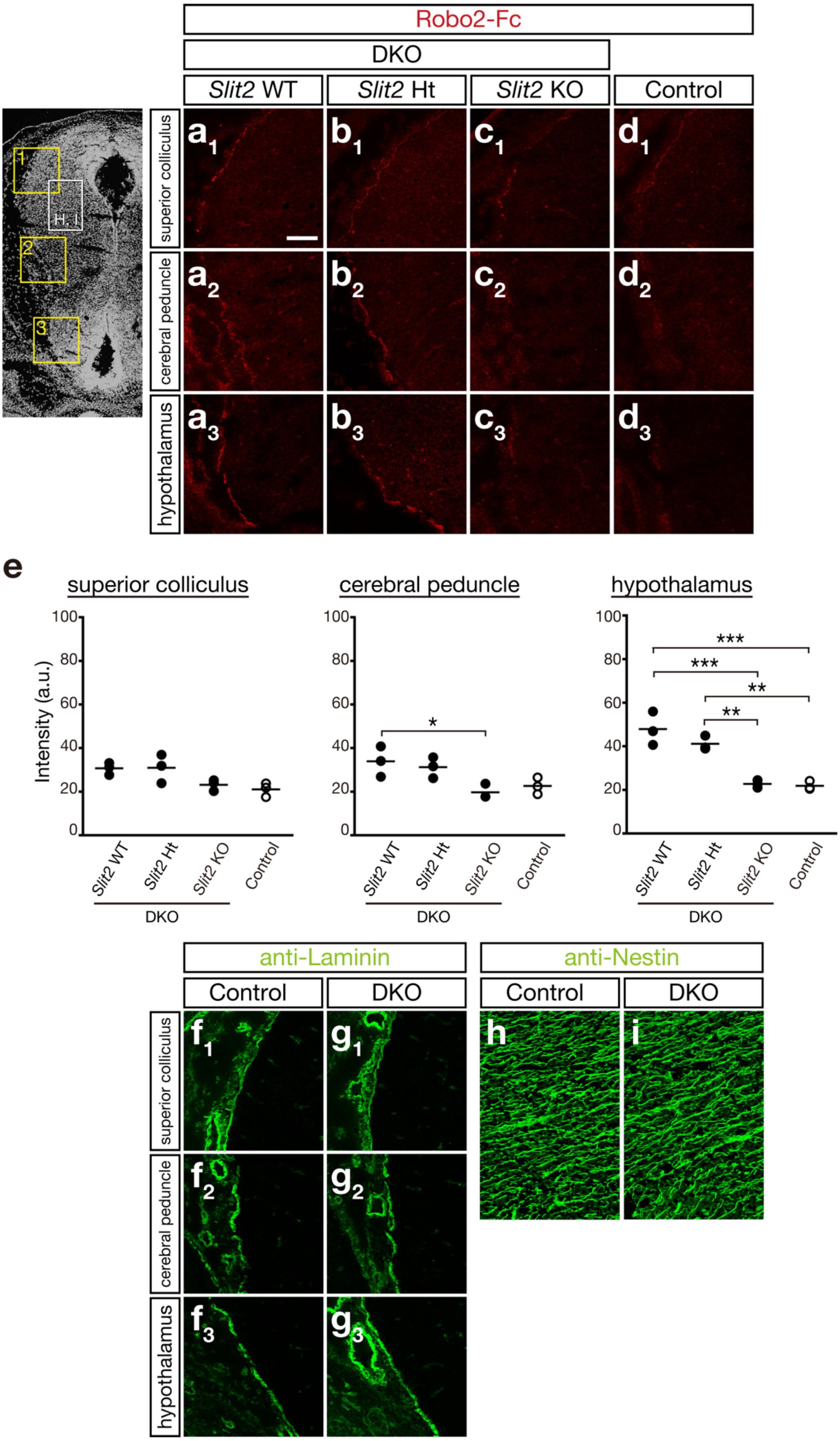
Robo2-Fc
DKO
Slit2 WT
Slit2 Ht
Slit2 KO
Control
superior colliculus
cerebral peduncle
hypothalamus
a1 b1 c1 d1
a2 b2 c2 d2
a3 b3 c3 d3
1
2
3
H, I
e
superior colliculus
cerebral peduncle
hypothalamus
Intensity (a.u.)
100
80
60
40
20
0
*
***
***
**
**
Slit2 WT
Slit2 Ht
Slit2 KO
Control
DKO
anti-Laminin
anti-Nestin
Control
DKO
f1 g1
f2 g2
f3 g3
h
i

Supplementary Figure S5. Robo2-Fc Binding in Slit2 Knockout Mice and Morphology of the Basement Membrane and Radial Glial Cells.

(**a**-**d**) Robo2-Fc binding on the brain sections of the *Sulf1/2* DKO mice that were wild-type (WT), heterozygous (Ht), or homozygous (KO) for *Slit2*, and that of the control mice (*Sulf1*$^{-/-}$;*Sulf2*$^{+/-}$) are shown. ($\mathbf{a_1}$-$\mathbf{d_1}$), ($\mathbf{a_2}$-$\mathbf{d_2}$), and ($\mathbf{a_3}$-$\mathbf{d_3}$) indicate the areas including the superior colliculus, cerebral peduncle, and hypothalamus, respectively.

(**e**) Quantitative analysis of Robo2-Fc binding. Average intensities of the fluorescence intensity (arbitrary unit; a.u.) on the pial surface obtained by confocal microscopy are shown. The Robo2-Fc binding in the *Sulf1/2* DKO mice was almost abolished by disruption of 2 alleles of *Slit2*, whereas disruption of 1 allele of *Slit2* did not significantly decrease the signal.

(**f**-**i**) E15.5 brains immunostained with anti-laminin (**f**, **g**) or anti-nestin (**h**, **i**) antibody. Control (**f**, **h**; *Sulf1*$^{-/-}$) and *Sulf1/2* DKO mouse brains (**g**, **i**) are shown. The morphology of the basement membrane and radial glial cells did not differ between the control and *Sulf1/2* DKO mouse brains. Pictures ($\mathbf{f_1}$, $\mathbf{g_1}$), ($\mathbf{f_2}$, $\mathbf{g_2}$), and ($\mathbf{f_3}$, $\mathbf{g_3}$) show high magnification of the boxed areas with the corresponding numbers in the brain section in the left margin, whereas pictures (**h**, **i**) show the area indicated by the white box.

Scale bars indicate 100 μm (**a**-**d**, **f**, **g**) and 65 μm (**h**, **i**).

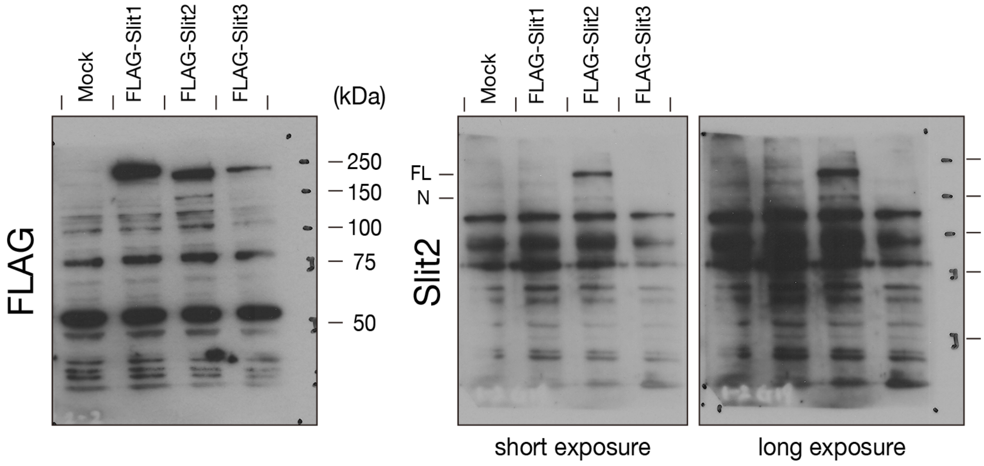

Supplementary Figure S6. Validation of Slit2 Antibody Specificity.

Cell lysates of COS-7 cells transfected with FLAG-Slit1, FLAG-Slit2, FLAG-Slit3, or vector alone (Mock) were subjected to Western blotting analysis. Slit1, Slit2, and Slit3 were detected by anti-FLAG antibody (left), whereas only Slit2 was detected by anti-Slit2 antibody (G-19; Santa Cruz Biotechnology; right). FL and N indicate the full-length and N-terminal fragments of Slit2, respectively. These data indicate specificity of the antibody among Slit proteins.

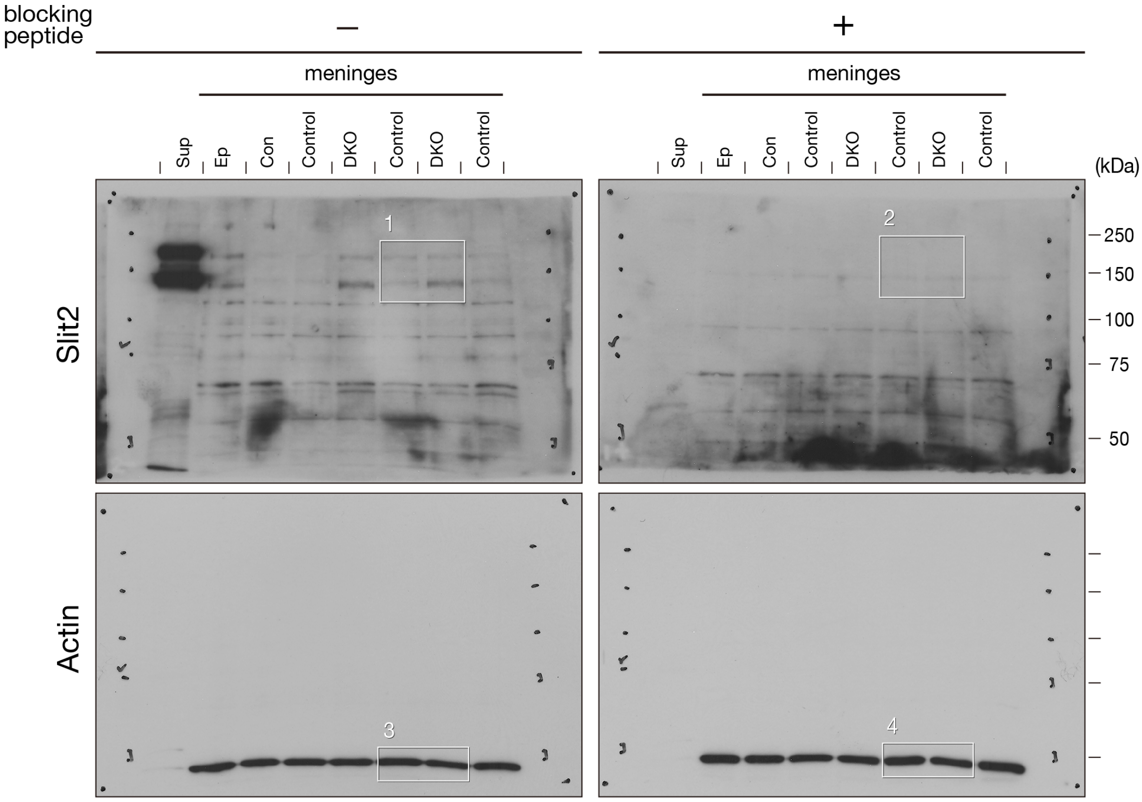

Supplementary Figure S7. Raw data of Western Blots.

Full-length blots that were used for Figure 5 are shown.

Supernatants from Slit2-expressing 293EBNA cells (a positive control; Sup), and lysates of meninges dissected from the hypothalamic and midbrain regions of E15.5 mouse embryos were subjected to Western blotting analysis. Signals were detected by anti-Slit2 antibody (upper columns; G-19; Santa Cruz Biotechnology), followed by stripping and detection by anti-actin antibody (lower columns). The same sample sets were detected by nontreated anti-Slit2 antibody (left columns) or the same antibody that had been preabsorbed with the blocking peptide (right columns). Ep and Con indicate the electroporated side and control side of the mouse embryos that had been electroporated with *Slit2*. Control and DKO indicate the lysates from the control and DKO mouse embryos. Boxed areas 1-4 are cropped and shown in Figure 5i.

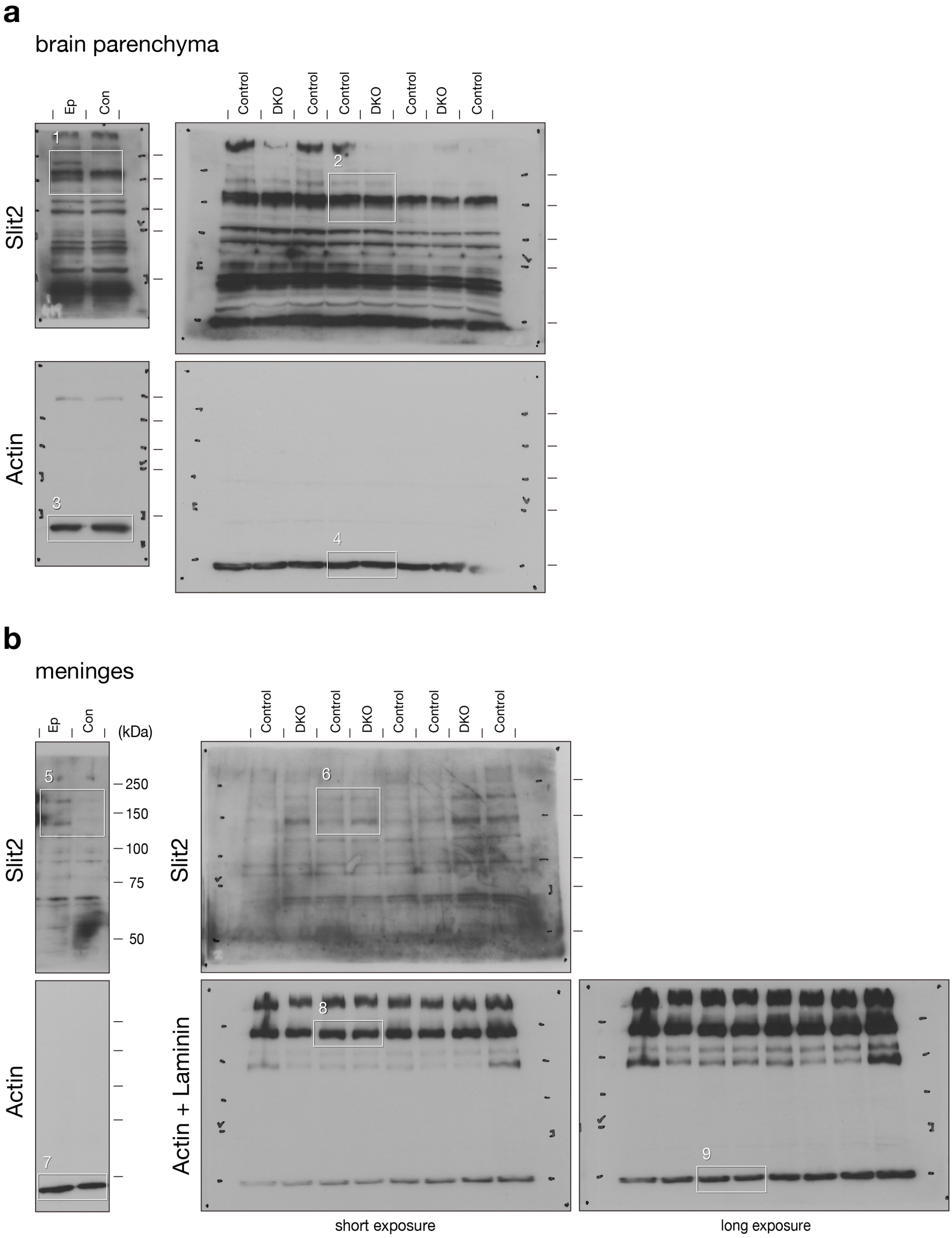
a
brain parenchyma
Ep
Con
Control
DKO
Control
Control
DKO
Control
DKO
Control
Slit2
Actin
1
2
3
4
b
meninges
Ep
Con
(kDa)
250
150
100
75
50
Control
DKO
Control
DKO
Control
Control
DKO
Control
Slit2
Actin
Actin + Laminin
5
6
7
8
9
short exposure
long exposure

Supplementary Figure S8. Raw Data of Western Blots.

Full-length blots that were used for Supplementary Figure S4 are shown.

Lysates of brain parenchyma (**a**) and meninges (**b**) dissected from the hypothalamic and midbrain regions of E15.5 mouse embryos were subjected to Western blotting analysis. Signals were detected by anti-Slit2 antibody (upper columns; G-19; Santa Cruz Biotechnology), followed by stripping and detection by anti-actin antibody alone or combination of anti-actin antibody and anti-laminin antibody (lower columns). Ep and Con indicate the electroporated side and control side of the mouse embryos that had been electroporated with *Slit2*, respectively. Control and DKO indicate the lysates from the control and DKO mouse embryos. Boxed areas 1-9 are cropped and shown in Supplementary Figure S4s. The cropped picture of the blot of the meningeal preparation of *Slit2*-electroporated brain in (**b**, left) is derived from the upper left blot in Supplementary Figure S7.

Supplementary Table 1. Statistical Analyses of the Neurofilament-Positive Fibers in the Midbrain

	n	Area (mm^2)
Control (Fig. 1i)	14	0.038 ± 0.004
DKO (Fig. 1k)	8	0.228 ± 0.01
P value (Welch's t-test)		4.12E-08****
DKO, pCX-Sulf1 + Sulf2	n	Area (mm^2)
Ep (Fig. 2q)	3	0.027 ± 0.008
Con (Fig. 2s)	3	0.233 ± 0.018
P value (Paired t-test)		0.01212*
DKO, pCX-Sulf1 (C87A)	n	Area (mm^2)
Ep (Fig. S2g)	3	0.221 ± 0.011
Con	3	0.231 ± 0.015
P value (Paired t-test)		0.58721
DKO, pCX-Sulf1	n	Area (mm^2)
Ep (Fig. S2k)	3	0.029 ± 0.002
Con	3	0.222 ± 0.02
P value (Paired t-test)		0.012999*

DKO, pCX-Sulf2	n	Area (mm^2)
Ep (Fig. S2o)	3	0.032 ± 0.01
Con	3	0.258 ± 0.029
P value (Paired t-test)		0.007525**
DKO, pEF-BOS-Sulf1 + Sulf2	n	Area (mm^2)
Ep (Fig. 3c)	3	0.034 ± 0.005
Con	3	0.198 ± 0.031
P value (Paired t-test)		0.026163*
WT, pCX-Sema3e	n	Area (mm^2)
Ep (Fig. 4i)	3	0.036 ± 0.007
Con	3	0.034 ± 0.005
P value (Paired t-test)		0.900968
WT, pEF-BOS-Slit2	n	Area (mm^2)
Ep (Fig. 4p)	3	0.233 ± 0.009
Con	3	0.032 ± 0.011
P value (Paired t-test)		0.000936***

We quantitated the signal of neurofilament-positive fibers in the whole-mount staining using Image J software (https://imagej.nih.gov/ij/) as described below. Briefly, the region of interest was defined by use of anatomical structures (dorsal is the lower margin of the optic tract in the superior colliculus; ventral is the upper margin of the cerebral peduncle; anterior is the posterior margin of the optic tract; posterior is the anterior margin of the inferior colliculus). The area (mm^2) of the portion in the demarcated region that exceeded the background threshold (defined as the highest value in the inferior colliculus) was measured. Statistical analysis was done using Welch's t-test and a paired t-test, and *P* values were obtained (*, $P < 0.05$; **, $P < 0.01$; ***, $P < 0.001$; ****, $P < 0.0001$). In the electroporation experiments (Figures 2q, 2s, 3c, 4i, 4p, S2g, S2k, and S2o), the values of the elecroporated side (Ep) and the control nonelectroporated side (Con) of the same mice were compared.
